# Unveiling the drives behind tetracycline adsorption capacity with biochar through machine learning

**DOI:** 10.1038/s41598-023-38579-8

**Published:** 2023-07-17

**Authors:** Pengyan Zhang, Chong Liu, Dongqing Lao, Xuan Cuong Nguyen, Balasubramanian Paramasivan, Xiaoyan Qian, Adejumoke Abosede Inyinbor, Xuefei Hu, Yongjun You, Fayong Li

**Affiliations:** 1 Key Laboratory of Tarim Oasis Agriculture (Tarim University), Ministry of Education, Xinjiang, 843300 China; 2 College of Water Resources and Architectural Engineering, Tarim University, Xinjiang, 843300 China; 3 College of Information Engineering, Tarim University, Xinjiang, 843300 China; 4 Institution of Research and Development, Duy Tan University, Da Nang, 550000 Vietnam; 5 Department of Biotechnology and Medical Engineering, National Institute of Technology Rourkela, Odisha, 769008, India; 6 Department of Physical Sciences, Industrial Chemistry Programme, Landmark University, Omu-Aran, Kwara State Nigeria

**Keywords:** Pollution remediation, Computer science

## Abstract

This study aimed to develop a robust predictive model for tetracycline (TC) adsorption onto biochar (BC) by employing machine learning techniques to investigate the underlying driving factors. Four machine learning algorithms, namely Random Forest (RF), Gradient Boosting Decision Tree (GBDT), eXtreme Gradient Boosting (XGBoost) and Artificial Neural Networks (ANN), were used to model the adsorption of TC on BC using the data from 295 adsorption experiments. The analysis revealed that the RF model had the highest predictive accuracy (R^2^ = 0.9625) compared to ANN (R^2^ = 0.9410), GBDT (R^2^ = 0.9152), and XGBoost (R^2^ = 0.9592) models. This study revealed that BC with a specific surface area (S (BET)) exceeding 380 cm^3^·g^−1^ and particle sizes ranging between 2.5 and 14.0 nm displayed the greatest efficiency in TC adsorption. The TC-to-BC ratio was identified as the most influential factor affecting adsorption efficiency, with a weight of 0.595. The concentration gradient between the adsorbate and adsorbent was demonstrated to be the principal driving force behind TC adsorption by BC. A predictive model was successfully developed to estimate the sorption performance of various types of BC for TC based on their properties, thereby facilitating the selection of appropriate BC for TC wastewater treatment.

## Introduction

Tetracycline (TC) is extensively employed as an antimicrobial agent and feed supplement in agriculture and animal husbandry^1^. However, researchers have recently paid significant attention to the issues of incomplete metabolism and TC emissions^2,3^. Due to its persistence as an organic pollutant, TC is frequently detected in surface water, groundwater, and drinking water. TC can induce endocrine disruption in target organisms and can also contribute to the dissemination of antibiotic resistance genes, thereby posing serious human health concerns and environmental hazards^4,5^. Given the inhibitory effect of TC on microorganisms, the removal of TC from water bodies using conventional biological water treatment methods proves challenging^6^.

Currently, the principal methods for treating TC in wastewater include chemical oxidation, biological treatment, and physical removal^2^. Adsorption, on account of its inherent advantages, such as simplicity, low cost, and high efficiency, is viewed as an excellent technology for the treatment of TC. Among the various adsorbents, biochar (BC) has been extensively researched as an adsorbent for removing pollutants from wastewater due to its unique characteristics, such as a large specific surface area, uniform pore distribution, and high concentration of surface functional groups^7^.

The uptake of tetracycline onto biochar mainly involves physical interactions such as van der Waals forces and hydrogen bonding, as well as chemical reactions including covalent and ionic bonding^8^. Therefore, the adsorption process primarily depends on the properties of biochar, adsorption conditions, and the ratio of adsorbate to absorbent. Several traditional kinetic and isothermal adsorption models have been extensively evaluated in previous studies^9–11^. Findings suggest that the possible adsorption mechanisms include π–π interactions, electrostatic interactions, and chemisorption. Although a typical controlled-variable experimental approach can determine the relationship between each influencing factor and the amount of sorption within the same framework, traditional batch sorption experiments are time-consuming and inefficient when selecting suitable biochar^12^. Therefore, there is an urgent need to develop practical tools for predicting adsorption efficiency, optimizing process parameters, and comprehending the adsorption mechanism.

Machine learning (ML)-assisted modeling has been proposed as a potential approach to reduce the cost and time associated with laboratory contaminant removal processes. Previous research has utilized machine learning (ML) algorithms on selected carbon-based materials to adsorb tetracycline (TC)^13,14^, yet the accuracy of the models could be enhanced. Zhu et al.’s^13^ study employed carbon-based materials such as activated carbon and biochar, which have distinct compositions. Thus, developing prediction models for both materials represents a significant challenge due to the high variability; in addition, the study has a limited database, and the highest achieved R^2^ value was only 0.8944, highlighting the necessity to optimize the machine learning (ML) model^15,16^. This study will also use the results of the machine learning model to explore the driving factors for the adsorption of tetracycline on biochar. To evaluate the prediction effectiveness, generalized adsorption models must be utilized to predict TC adsorption on a single biochar, particularly integrated learning models. Ensemble learning is a typical ML algorithm that integrates the modeling outcomes of all models by building multiple models from the data^17^. The most typical ensemble learning algorithms used in assessing TC adsorption on biochar include random forest (RF), gradient boosting decision tree (GBDT), and eXtreme gradient boosting (XGBoost)^18^. In addition to ensemble learning, this study will also incorporate the most popular deep learning algorithms as a point of comparison.

The integration of machine learning as an advanced algorithm within the field of environmental remediation employing biochar remains in its nascent stage, considering the widespread occurrence, substantial ecological risk, and unique properties of toxic compounds (TC) in the environment. This research was conducted with the aims of: (i) devising universal machine learning models to forecast the sorption capacity of TC on biochar (BC), contingent on BC attributes and sorption conditions; (ii) investigating the primary factors contributing to BC adsorption of TC; (iii) assessing the impact of various factors on the relative significance of BC sorption capacity and ascertaining the combined effect of each factor on BC sorption capacity; and (iv) constructing an accessible web-based user interface for engineers. The machine learning-driven model devised in this investigation establishes a theoretical foundation for the pragmatic treatment of TC, delivering an all-encompassing comprehension of TC sorption on biochar relative to its features and sorption milieu.

## Materials and methods

### Cum biochar sorption capacity predictions layout

Experimental data for the adsorption of tetracycline by biochar were collected from ten papers, including 22 biochar species and 295 sets of experimental adsorption data^9,19–28^. Without author bias, the related articles were selected randomly and data were extracted from published papers using Plot Digitizer v3 (https://plotdigitizer.com/#download)^29^. Detailed data are provided in the supplementary materials (Tables S1, S2).

To predict the sorption capacity of BC for TC, expressed as the equilibrium sorption capacity Q_e_ (mg·g^−1^), 12 critical factors were considered and divided into three categories: (i) biochar properties: Brunauer–Emmett–Teller surface area [S (BET), m^2^·g^−1^], pH of the biochar in water (pH_H_2_O), total carbon in the biochar (C, w%), molar ratio of oxygen and nitrogen to carbon [(O + N)/C], molar ratio of oxygen to carbon (O/C), molar ratio of hydrogen to carbon (H/C), ash content (Ash, w%), pore volume (V, cm^3^·g^−1^), and biochar pore diameter (D, nm); (ii) adsorption conditions: adsorption temperature (T, °C) and solution pH (pH_sol); and (iii) initial concentration ratio of tetracycline to biochar (C_0_, mmol·g^−1^). In the ML section, data not provided in the published paper were replaced with K-Nearest Neighbor (KNN) Algorithm, while pH_H_2_O and ash were missing more often than the other values and was decided to remove them. The TC characteristics are listed in Table S3.

The following equation was used to obtain *C*_*0*_^30^:

1 $$\begin{array}{c}{\text{C}}_{0}\text{=} \, \frac{{\text{C}}_{\text{TC}}/444.4}{{\text{C}}_{\text{BC}}},\end{array}$$

where ${\text{C}}_{\text{TC}}$ (mg·L^−1^) is the initial concentration of TC and ${\text{C}}_{\text{BC}}$ (g·L^−1^) is the initial concentration of BC.

### Pre-processing of data

The linear correlation between any two randomly selected variables or between variables and target values was measured using the Pearson correlation coefficient using the following equation^30,31^:

2 $$\begin{array}{c}{r}_{xy} = \frac{{\sum }_{i=1}^{n}\left({x}_{i}-\overline{x }\right){\sum }_{i=1}^{n}\left({y}_{i}-\overline{y }\right)}{\sqrt{{\sum }_{i=1}^{n}{\left({x}_{i}-\overline{x }\right)}^{2}}\sqrt{{\sum }_{i=1}^{n}{\left({y}_{i}-\overline{y }\right)}^{2}}}, \end{array}$$

where $\overline{x }$ and $\overline{y }$ denote the mean of variable x or y.

### Construction of ML models

*Ensemble learning* is a popular machine-learning algorithm that integrates multiple models (base estimators) to form an ensemble estimator that solves complex problems with specific rules^17,32^. Integrated models act as an integrated platform to automatically manage the weaknesses and enhancements of individual models to achieve higher prediction accuracy. Three integration algorithms exist: bagging, boosting, and stacking.

RF is a representative bagging integration algorithm and is the most commonly used algorithm for predicting poorly understood processes^33^. The outcome of an RF prediction is a combination of the predicted outcomes of each decision tree, so the critical step in Random Forest prediction is the formation of a decision tree and a forest. This principle is depicted in Fig. S1.

The Gradient Boosted Decision Tree (GBDT) algorithm, an iterative decision tree algorithm, consists of multiple decision trees, and the conclusions of all the trees are summed to arrive at the final answer. As shown in Fig. S2, the GBDT algorithm uses the negative gradient value of the loss function of the base model in round i as an approximation of the loss value of the base model in that round^34^. The next step is to construct round i + 1 of base models based on this approximation to make the solution of the objective function more convenient^34^.

XGBoost is an improved version of GBDT. It has an engineering goal of pushing the computational power of boosting trees to a limit to achieve fast computation and superior performance^35^. With many improvements over traditional gradient boosting algorithms, XGBoost can be performed faster than other comprehensive algorithms that use gradient boosting and is recognized as an advanced evaluator with ultrahigh performance in both classification and regression.

In this study, Artificial Neural Network (ANN) was utilized due to its ability to simulate the connections and signal propagation between neurons, allowing the adjustment of weights using the backpropagation algorithm to learn patterns and relationships in the data. ANN consists of an input layer, hidden layers, and output layer, making it suitable for various tasks such as prediction, classification, and pattern recognition.

All machine-learning algorithm codes were obtained from the open-source Scikit-learn library. All datasets were divided into training and test data at a ratio of 70:30, with the random state set to 40. Tenfold cross-validation was used to select the best hyperparameters from the data. The test data were used to evaluate model performance. All data were normalized before training. All input and output parameters in this study are listed in Table S4.

### Modeling performance evaluation

The performance of the model was assessed using the coefficient of determination (R^2^) and the root mean squared error (RMSE)^13,30,35^.

3 $$\begin{array}{c}{R}^{2}=1- \frac{{\sum }_{i=1}^{N}{\left({Y}_{i}^{exp}- {Y}_{i}^{pred}\right)}^{2}}{{\sum }_{i=1}^{N}{\left({Y}_{i}^{exp}- {Y}_{ave}^{exp}\right)}^{2}},\end{array}$$

4 $$\begin{array}{c}RMSE= \sqrt{\frac{1}{N}\sum_{i=1}^{N}{\left({Y}_{i}^{exp}-{Y}_{i}^{pred}\right)}^{2},}\end{array}$$

where ${Y}_{i}^{exp}$ and ${Y}_{i}^{pred}$ are the experimental and predicted values, and ${Y}_{ave}^{exp}$ is the average of the experimental values.

## Results and discussion

### Statistical results of biochar characteristics

**Figure 1:**
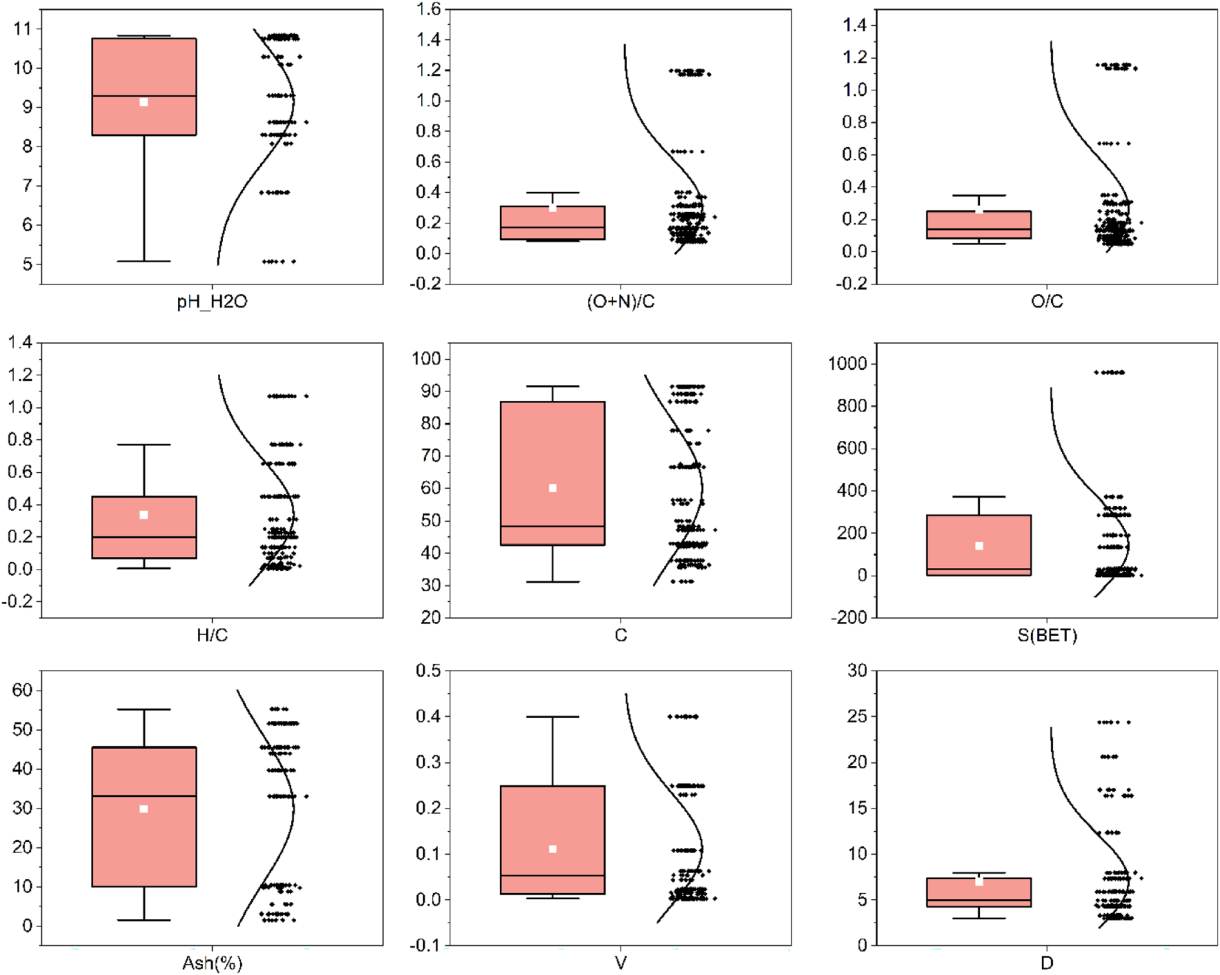
Visualization of biochar properties through box plots and normal distribution curves.

This study utilized a combination of box plots and normal distribution curves to illustrate the distribution patterns of continuous data (see Fig. 1). The composite plot comprises two sections—the left segment illustrates the box plot, whereas the right segment manifests the normal distribution curve of the data. The box plot depicts the median, signified by a white dot, the interquartile range, denoted by the box, and the whiskers, which represent the remaining data. Outliers are designated by circular points or alternative symbols. The probability density of data at each value is displayed on the right portion of the plot, with elevated values indicating a comparatively higher probability of data occurrence at that point. Please refer to Table S5 for specific values.

The majority of biochars exhibit alkalinity in water, a phenomenon predominantly correlated with the depletion of acidic functional groups and an augmentation in aromatic carbon at elevated temperatures, but also linked to the accumulation of alkaline ions such as Na^+^, Ca^2+^, K^+^, and Mg^2+^ in the biochar^30^. The mean pH of all biochar samples was 9.14. Nevertheless, a minority displayed weak acidity, which can be accounted for by the incomplete liberation of alkali salts from the biochar matrix at lower pyrolysis temperatures^36,37^. Given that pH exerts a significant impact on the adsorption of tetracycline onto biochar, the pH of the solution was adjusted using either an acid or a base in all experiments selected for this investigation. Consequently, subsequent analyses will disregard the pH of the biochar in an aqueous solution (pH_H_2_O) and instead utilize the pH of the solution (pH_sol) during batch adsorption studies.

As depicted in Fig. 1, biochar exhibits a high carbon content, with an estimated average of approximately 60% and a maximum value of 92%. Existing research has demonstrated that the carbon content escalates in correlation with increasing pyrolysis temperatures within a specified range^20,23,38^. Consequently, the pyrolysis process concentrates carbon within the biomass feedstock^39,40^. The H/C, O/C, and (N + O)/C ratios serve as indicators of aromaticity, hydrophilicity, and polarity indices, respectively^39,40^. The H/C, O/C, and (N + O)/C ratios indicate the aromaticity, hydrophilicity, and polarity indices, respectively^41^. A lower H/C ratio in BC corresponds to higher aromaticity; a lower O/C ratio indicates reduced hydrophilicity; and an elevated (N + O)/C ratio signifies increased polarity^42,43^. The median O/C and H/C ratios were 0.14 and 0.2, respectively. According to the International Biochar Initiative (IBI) Standards, the H/C ratio for biochar should be less than 0.7. Therefore, values exceeding 0.7 in the dataset can be eliminated to enhance the accuracy of biochar data analysis. Nevertheless, Table S2 contains only limited data.

It has been posited that an increased S (BET) is advantageous for TC adsorption onto biochar, while D and V exhibit minimal direct influence, though an optimal range for each exists. The ash content of biochar samples exhibited a broad range, spanning from 1.50% to 55.27%, attributable to variations in feedstock type and pyrolysis conditions, which alter the physicochemical properties and spatial distribution of organic matter^30^. However, the role of ash in TC adsorption onto biochar remains a subject of debate.

### Statistical outcomes of data correlation analysis

**Figure 2:**
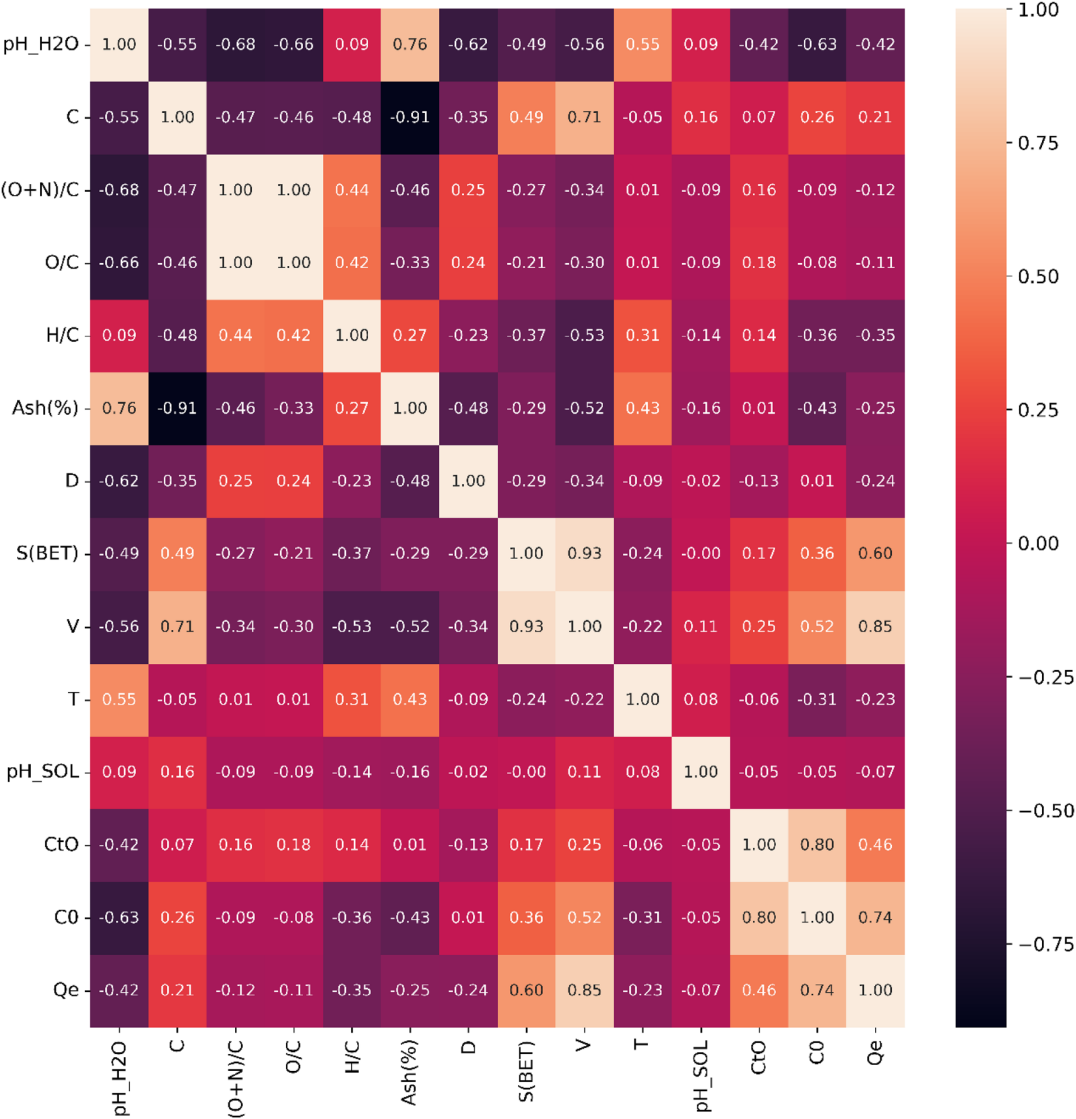
Correlation coefficients and corresponding significant levels.

Figure 2 demonstrates that there exists a significant positive correlation between C_0_, S (BET), and V with respect to Qe. This relationship can be elucidated by the transfer equation: when the adsorption value remains constant, an increase in the amount of adsorbent leads to a higher adsorption capacity per unit mass of adsorbed material. Research conducted by Wang et al., Zhu et al., and Kim et al.^20,23,30^ substantiates the connection between S (BET) and Qe, suggesting that an elevated specific surface area permits more adsorbates to be adsorbed per unit mass of the adsorbent. Additionally, a higher number of pores per unit of adsorbent contributes to an increased adsorption capacity, as evidenced by the correlation between V and Qe.

Moreover, in accordance with prior research findings^30^, S (BET) displays a positive correlation with carbon content, yet a negative correlation with ash content. This observation implies that elevated carbon content results from the removal of volatile substances, while a higher ash content can cause micropore filling, consequently reducing the surface area^6,13^. Furthermore, the inverse correlation between S (BET) and D lends support to previous studies suggesting that an increase in S (BET) corresponds to a decrease in D. The correlation coefficient between ash and carbon was − 0.91, and the correlation coefficients between (O + N)/C and O/C were 1. Thus, one variable from each pair must be excluded to prevent collinearity.

### Selection of machine models for tetracycline adsorption on biochar

**Figure 3:**
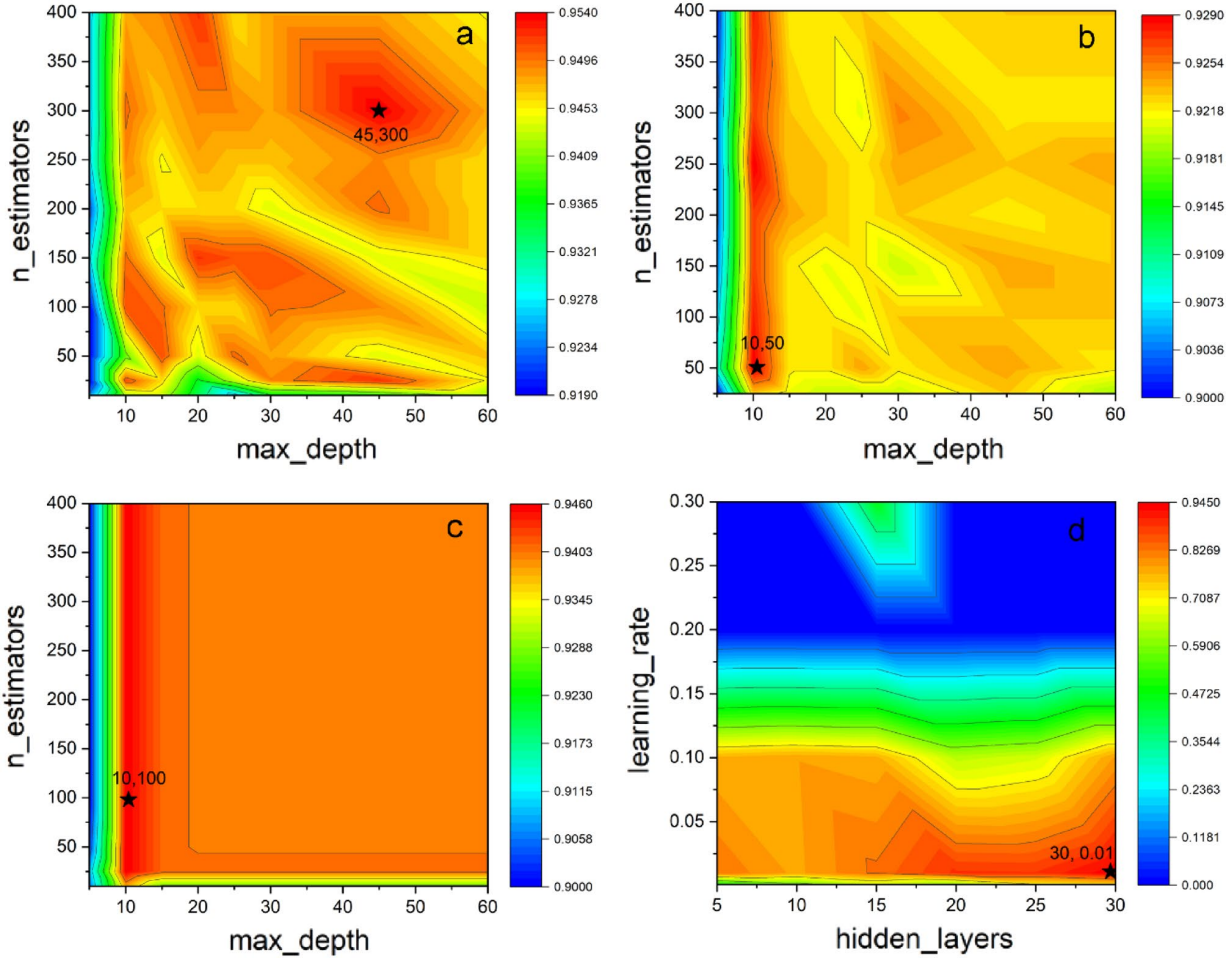
Schematic illustration of hyperparameter selection (**a** RF, **b** GBDT, **c** XGBoost, **d** ANN).

In order to assess the potential of machine learning in predicting the adsorption of tetracycline on biochar, a tenfold cross-validation was executed to determine hyperparameters. Figure 3 depicts the two most significant parameter combinations, ‘n_estimators’ and ‘max_depth’ (‘hidden layer’ and ‘learning rate’) with the color on the surface model representing the model’s performance quality. The redder the color, the higher the model’s accuracy, as per the hyperparameter selection principle. The parameters selected in this study were determined to be equal to or greater than the threshold value of two.

**Figure 4:**
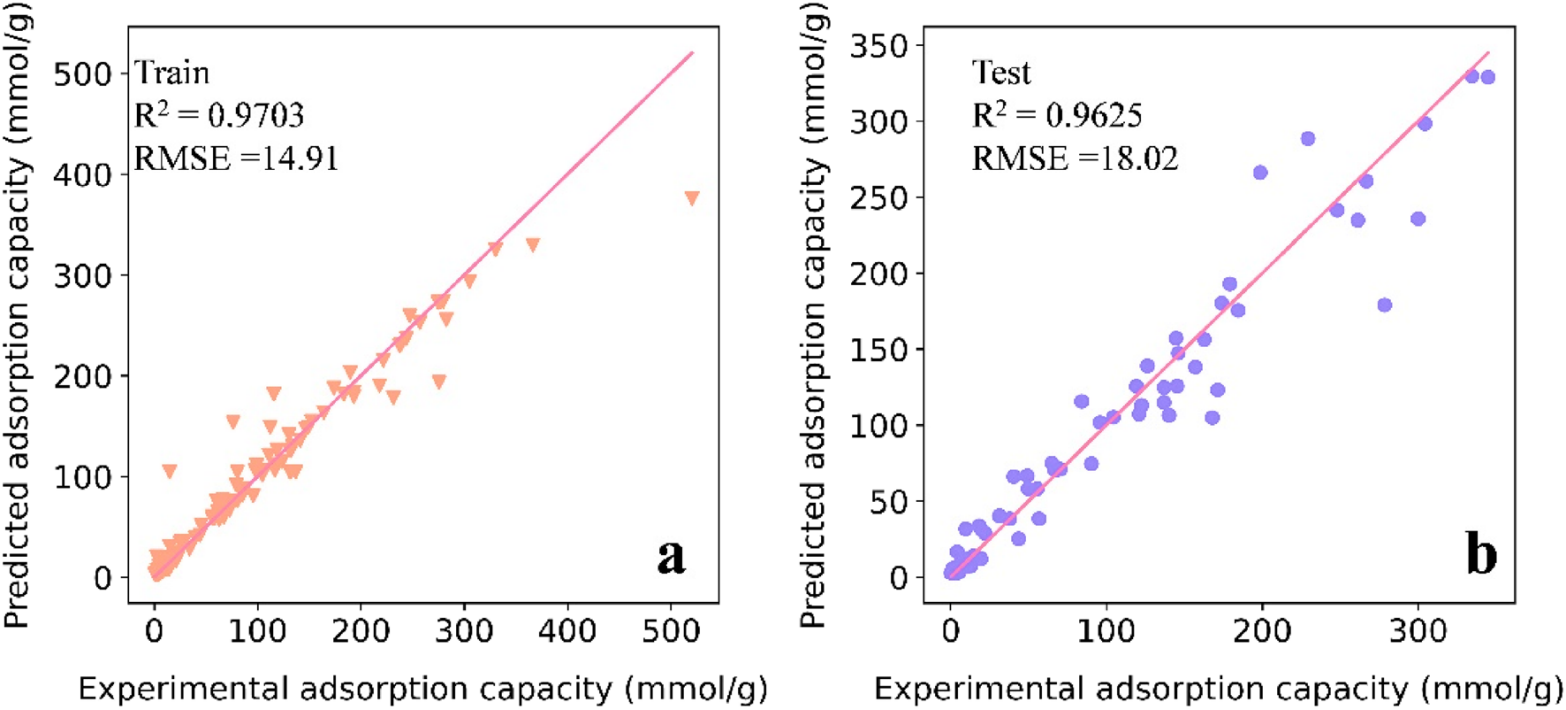
Scatter plot of RF-model-predicted adsorption values and experimental data (**a** training data, **b** testing data).

As shown in Table S4, the highest mean R^2^ value of 0.9625 was achieved by Random Forest (RF), followed by XGBoost at 0.9592, while Gradient Boosted Decision Trees (GBDT), had the lowest score of 0.9152, and the artificial neural network (ANN) had the second-to-last score of 0.9410. The RMSE values for RF, GBDT, ANN, and XGBoost were 18.02, 25.97, 22.42, and 20.99, respectively. As illustrated in Fig. 4, both the training and test sets exhibited R^2^ and RMSE values of 0.9703, 0.9625, and 14.91, and 18.02, respectively, for RF and XGBoost. The model did not appear to be overfitted. Although XGBoost is generally regarded as the superior model, in this study, RF outperformed XGBoost. One possible explanation is that the results were influenced by the features and the nature of the problem. Therefore, while XGBoost is among the better models, it may not always be the optimal choice, exemplifying the “no free lunch theorem”.

According to Fig. 3, the predicted values for all data ranged from 0 to 350 mmol·g^−1^. The RMSE of the RF model (18.02 mmol·g^−1^) accounted for 0.0542 of the predicted range (0 to 350 mmol·g^−1^), indicating a high level of accuracy. Combining the accuracy of R^2^ and RMSE, the RF model was selected for subsequent analysis since it provided higher accuracy than the XGDT and XGBoost models. The best parameters for each model are listed in Table S4.

### Feature importance analysis

This section is based on the RF model. Feature importance analysis serves as a potent instrument for discerning the relevance of input features in target prediction. Employing machine learning to comprehend tetracycline (TC) adsorption on biochar (BC) can substantially curtail the time-consuming and costly experimental design process by leveraging feature importance to select a limited number of features for model training, thereby reducing time and cost while enhancing accuracy^44,45^. Although machine learning models are formidable tools for generating precise predictions, they frequently function as “black box” models, rendering the comprehension of their inner mechanisms and decision-making processes arduous. Nevertheless, feature importance analysis offers an efficacious approach for pinpointing the most crucial input variables in a model and comprehending their contributions to the output.

**Figure 5:**
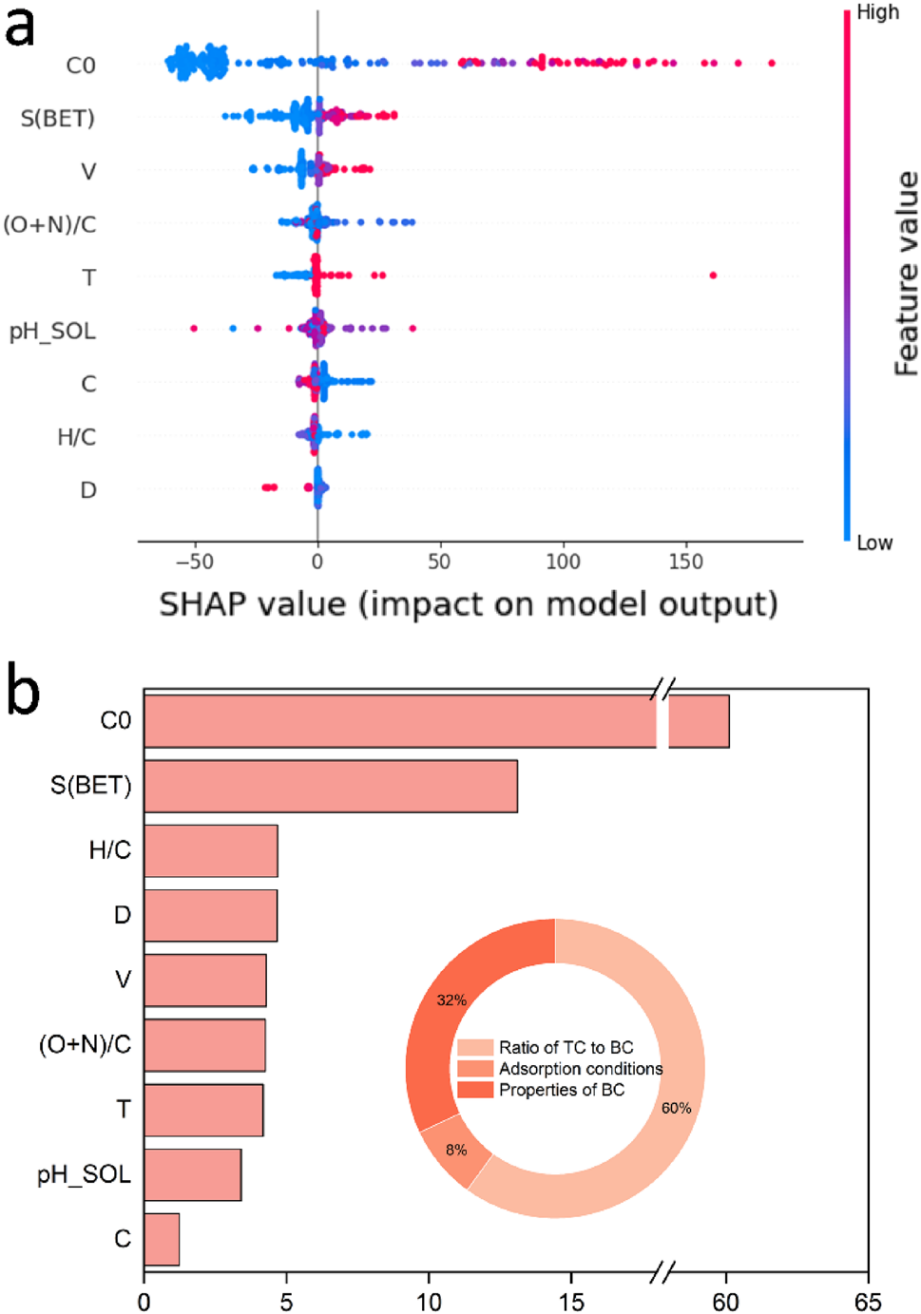
Relative importance of input variables on sorption capacity using SHAP (**a** SHAP value; **b** feature importance).

A primary advantage of employing SHAP (SHapley Additive exPlanations) for feature importance analysis is its ability to furnish a visual representation of each feature’s contribution to the output prediction. Figure 5a exhibits a SHAP feature importance visualization, supplying an in-depth dissection of the weight and influence exerted by each input feature on the predicted outcome. This information can be harnessed to identify vital control parameters, optimize the experimental design process, and bolster model accuracy.

Figure 5b displays the specific values of feature importance derived from SHAP. The results suggest that C_0_ (0.695) is the most critical factor affecting Qe, signifying that BC’s adsorption capacity for TC predominantly depends on the TC-to-BC ratio. This phenomenon, referred to as the value transfer process, implies that the concentration gradient between the adsorbate and the adsorbent constitutes the principal driving force for TC adsorption by BC.

In examining the biochar characteristics, surface area (S (BET)), (O + N)/C, and H/C ratios displayed notably significant effects (0.162, 0.036, and 0.032, respectively), suggesting that S (BET) is the most crucial factor influencing biochar’s adsorption properties. It can be deduced that the sites provided by S (BET) also play a vital role as driving factors in the adsorption of target compounds (TC) by biochar (BC). Concerning adsorption conditions, temperature (T) and pH_sol contributed to 0.14 and 0.06 of the characteristic importance, respectively. The impact of each factor on Qe is discussed in “Analysis of partial dependence plots (PDP)” section.

### Analysis of partial dependence plots (PDP)

**Figure 6:**
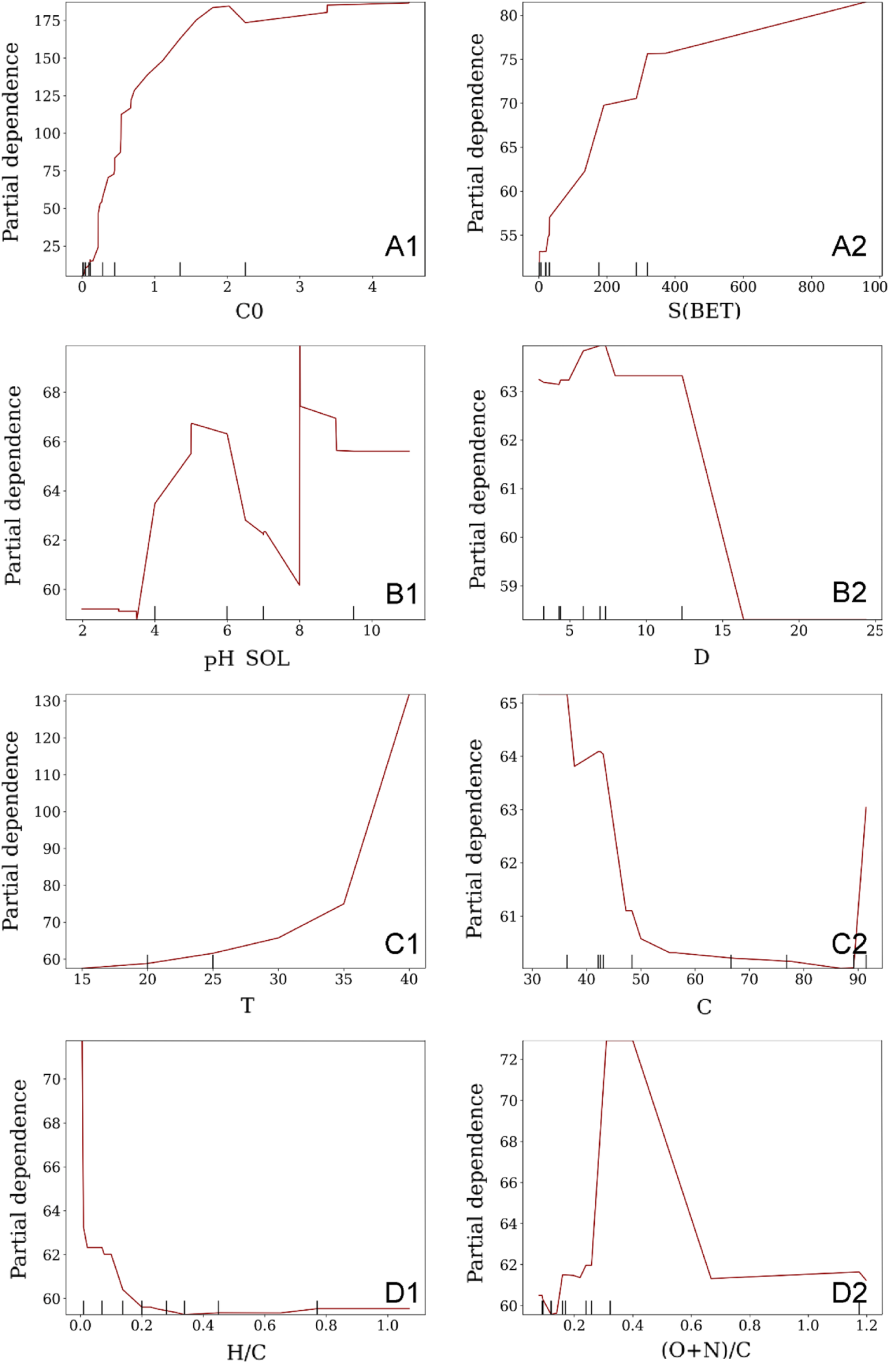
The PDP of TC adsorption on significant variables (spikes on the x-axis represent data density).

Figure 6(A1) presents the single-factor PDP, which reveals a partial dependence of the initial concentration (C_0_) on the equilibrium adsorption capacity (Qe), demonstrating an initial increase in the adsorption rate followed by stabilization. This trend can be attributed to the gradual filling of adsorption sites on the biochar surface as the relative content of TC increases. Upon reaching C_0_ = 2 mmol·g^−1^, the adsorption sites on the biochar surface become fully occupied, resulting in maximum adsorption capacity. The saturation of adsorption sites limits any further increase in the removal rate. These findings highlight the critical role of biochar surface area and capacity in effective pollutant removal^21^.

Biochar’s adsorption efficacy is contingent upon its specific surface area (S (BET)). As depicted in Fig. 6(A2), a rapid rise in adsorption capacity is observed, followed by a gradual increase. The enhancement in the number of sorption sites with an increase in S (BET) leads to a higher sorption uptake capacity of biochar. Nonetheless, the adsorption efficacy of biochar is constrained by other factors, such as the initial concentration of the target compound (C_0_), when there is a certain increase in the number of adsorption sites^20,21^. S (BET) predominantly affects the driving force of chemisorption occurring between BC and TC. A larger S (BET) results in a more significant number of chemisorption sites, thus leading to higher adsorption efficiency. Furthermore, the indirect influence of biochar’s specific surface area on physical adsorption should not be overlooked. The adsorption of the target compound (TC) on biochar is the result of various driving forces. Based on Fig. 6(A2), it can be inferred that biochar with an S (BET) greater than 380 cm^3^·g^−1^ offers superior adsorption efficiency.

The distribution of tetracycline (TC) species is influenced by the pH value. Table S3 presents the dissociation constants (pKas) of TC as 3.3, 7.7, and 9.7. Within the pH range of 2–3.3, TC^+^ is the predominant form of TC; at pH 3.3–7.7, TC_0_ is prevalent; at pH 7.7–9.7, TC^−^ dominates, and at pH above 9.7, TC converts to TC^2−^. Figure 5(B1) illustrates that the adsorption of TC on biochar (BC) is most favorable when the solution pH (pH_sol) is approximately 5.5. Intriguingly, these findings contradict a report by Ref.^21^, which posited that biochar and tetracycline primarily undergo electrostatic adsorption. Despite the biochar’s negative charge, it assumed a positive charge when the pH ranged between 3 and 3.3. Figure 6(A3) indicates that, in addition to electrostatic adsorption, hydrogen bonding and π–π electron donor–acceptor interactions influence TC adsorption on biochar^19–21^.

The adsorption efficacy of biochar is also partially dependent on the D value. Figure 6(B2) indicates that adsorption efficiency increases, stabilizes, and then significantly decreases. Biochar with a D value of 2.5–14.0 nm demonstrates a higher tendency to adsorb TC. Nonetheless, this change is insignificant when compared to other factors. This study affirms the findings of Zhang et al.^21^ that the adsorption performance of adsorbents is optimal when the molecular size of the adsorbent’s D is 1.7–3.0 times larger than that of the adsorbate. However, these outcomes should be interpreted with caution.

Data for this investigation were gathered at temperatures between 15 and 40 °C (Fig. 6(C1)), with temperature fluctuations displaying minor, partially dependent variations from 15 to 35 °C. The adsorption efficiency’s partial dependence on temperature increases substantially within a higher temperature range of 35–40 °C, which aligns with previous findings that the adsorption process is thermodynamically favorable at elevated temperatures^6,24,25^. This phenomenon may be attributed to the diffusion of TC and enhanced interfacial chemistry.

The adsorption efficiency’s partial dependence on the chemical composition factors C, H/C, and (O + N)/C is relatively insignificant (Fig. 6(C2–D2)) and exhibits considerably less variation compared to changes in other factors. Consequently, this aspect will not be further explored in the present study.

**Figure 7:**
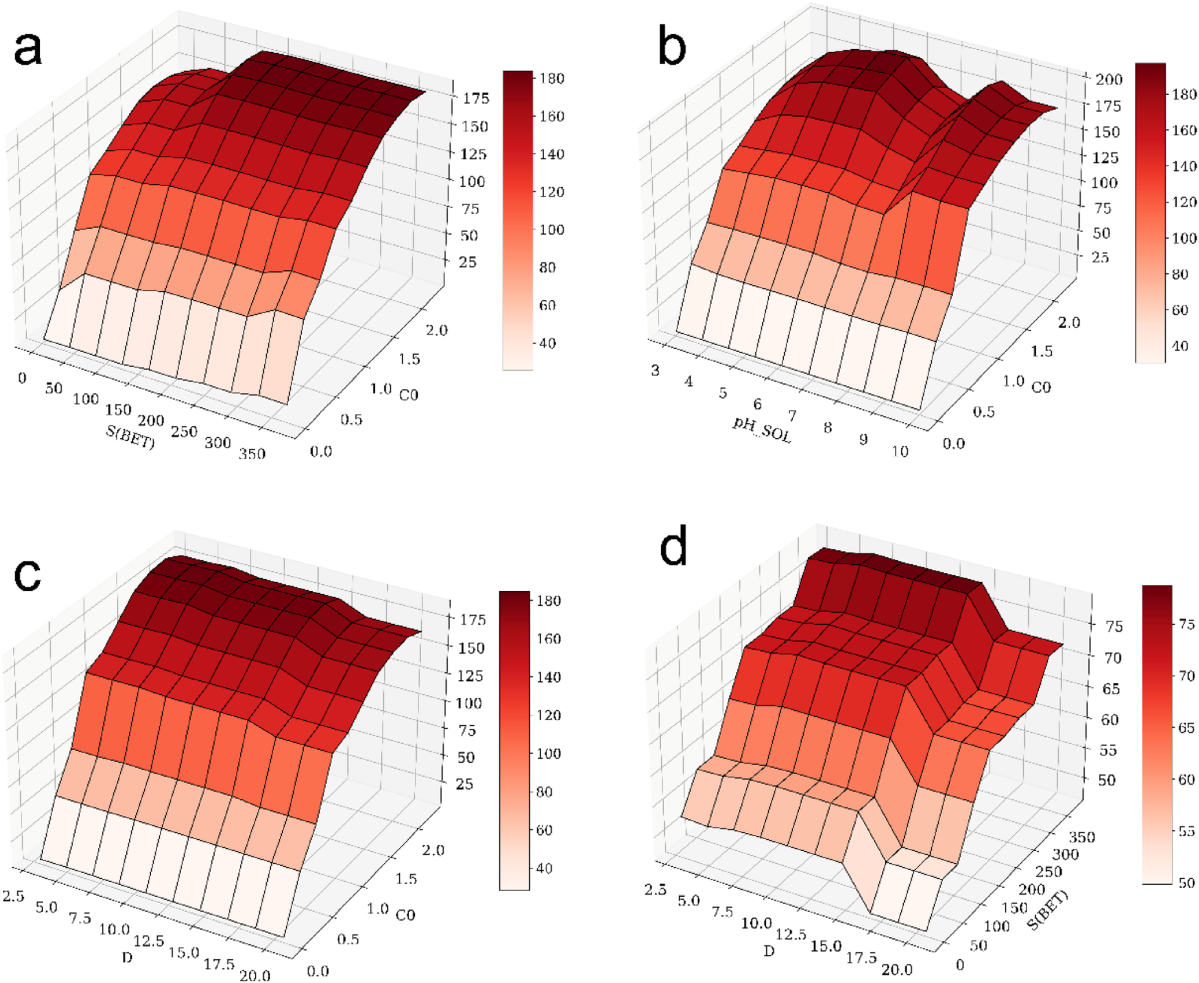
Bivariate PDP of TC adsorption on any two significant input variables and the interaction between the two variables.

A two-factor analysis was also employed to investigate the impact on TC adsorption, a representative depiction of which is provided in Fig. 7. (i) At a fixed specific surface area (S (BET)), the partial dependence increases with initial concentration (C_0_) and tends to stabilize when C_0_ > 2 mmol·g^−1^. (ii) The effect of pH_sol was less pronounced when comparing the partial dependences of C_0_ and pH_sol. (iii) At a fixed C_0_, the partial dependence tends to rise sharply when the temperature exceeds 35 °C.

### Significance and drawbacks of this study

The investigation of biochar preparation for tetracycline (TC) adsorption employing cost-effective biomass has recently emerged as a focal point of research, owing to its robust capacity to eliminate organic contaminants from aqueous media. Typically, controlled-variable experimental methodologies are employed to ascertain factors such as material characteristics and adsorption conditions. Nonetheless, conventional batch experiments are labor-intensive, expensive, and lack generalizability. In this study, Random Forest (RF) was demonstrated as a beneficial machine learning (ML) instrument for predicting the quantity of TC adsorbed by biochar, thereby showcasing its potential to directly forecast experimental outcomes based on pre-established conditions. Moreover, discerning the most crucial factors impacting TC sorption and their influence on the process offers invaluable insights for selecting or devising TC removal techniques. Consequently, the requisite number of experiments can be considerably diminished, and the exploration of biochar (BC) applications for TC adsorption can be markedly expedited.

This research revealed that the RF algorithm provides a reasonable prediction of TC adsorption quantities by biochar. However, the predictive performance of ML was hindered by data imbalance and scarcity, and several considerations must be addressed. The data utilized for model training solely predicted the sorption of TC by biochar, without accounting for the sorption of other antibiotics by the same material.

## Conclusion

This study successfully established a model for biochar adsorption of tetracycline (TC) using a comprehensive learning-based approach. The principal findings are as follows:

1. The Random Forest (RF) algorithm proved to be an accurate and effective predictor of TC adsorption by biochar (BC), achieving a coefficient of determination (R^2^) value of 0.9625, slightly outperforming the GBDT, ANN, and XGBoost algorithms.
2. The ratio of tetracycline to biochar significantly influenced the sorption process, with a weight of 0.595.
3. The primary driving force for the adsorption of TC by BC is the concentration gradient between the adsorbate and the adsorbent.
4. Biochar with initial concentration (C_0_) greater than 2 mmol·g^−1^, specific surface area (S (BET)) exceeding 380 cm^3^·g^−1^, and adsorbent diameter (D) ranging from 2.5 to 14.0 nm exhibited the highest propensity for adsorbing TC.

The model developed in this study has significant implications for minimizing redundant experimentation and facilitating the selection of appropriate biochar. Moreover, it will guide the proper application of biochar in tetracycline wastewater treatment technologies.

## Supplementary Information

Supplementary Information.

## Data Availability

All data generated or analysed during this study are included in this published article and its Supplementary Information files.

## Abbreviations

ML: Machine learning
RF: Random forest
RMSE: Root mean square error
PCC: Pearson correlation coefficient
GBDT: Gradient boosting decision tree
XGBoost: XExtreme gradient boosting
D: Particle size
S (BET) (m^2^·g^−1^): Brunauer–Emmett–Teller surface area
V (cm^3^·g^−1^): Total pore volume
C_0_ (mmol·g^−1^): Initial concentration ratio of tetracycline to biochar
Q_e_ (mg·g^−1^): Equilibrium adsorption capacity of tetracycline on biochar
TC: Tetracycline
BC: Biochar
PDP: Partial dependence plots
C: Total carbon in the biochar
pH_H_2_O: pH of the biochar in water
pH_sol: Solution pH
(O + N)/C: Molar ratio of oxygen and nitrogen to carbon
H/C: Molar ratio of hydrogen to carbon
Ash: Ash content
ANN: Artificial neural networks
