# Supplementary material for: Unveiling the drives behind tetracycline adsorption capacity with biochar through machine learning
Source: Sci Rep. 2023 Jul 17;13:11512. doi: 10.1038/s41598-023-38579-8 (PMC10352365; doi:10.1038/s41598-023-38579-8)
Supplement: Supplementary file 1 — Supplementary Information. [file 41598_2023_38579_MOESM1_ESM.docx]

**Supplementary Material:**

**Unveiling the Drives Behind Tetracycline Adsorption Capacity with Biochar through Machine Learning**

**Pengyan Zhang^1^,** **Chong Liu^1^, Dongqing Lao^2*^**, **Xuan Cuong Nguyen^3^, Balasubramanian Paramasivan^4^, Xiaoyan Qian^1^, Adejumoke Abosede Inyinbor^5^，Xuefei Hu^1^, Yongjun You^1^, Fayong Li^1^.**

^1^ *College of Water Resources and Architectural Engineering, Tarim University, Xinjiang 843300, China*

^2^ *College of Information Engineering, Tarim University, Xinjiang 843300, China*

^3^*Institution of Research and Development, Duy Tan University, Da Nang 550000, Vietnam*

^4^ *Department of Biotechnology and Medical Engineering, National Institute of Technology Rourkela, Odisha-769 008, India*

^5^ *Department of Physical Sciences, Industrial Chemistry Programme, Landmark University, P.M.B.1001, Omu-Aran, Kwara State, Nigeria*

*Corresponding author: Dongqing Lao

E-mail: 120100054@taru.edu.cn; Tel: 86-0997-4680383

Number of Figures: 2

Number of Tables: 6


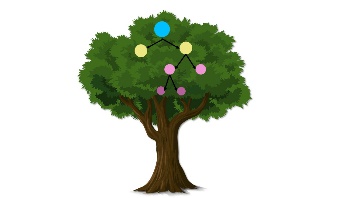

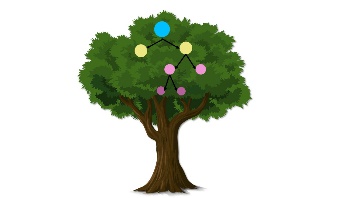

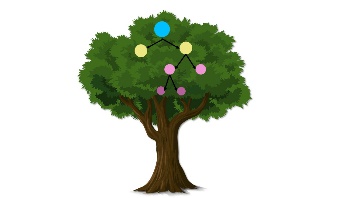

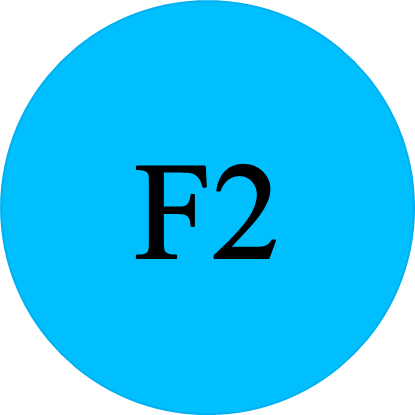

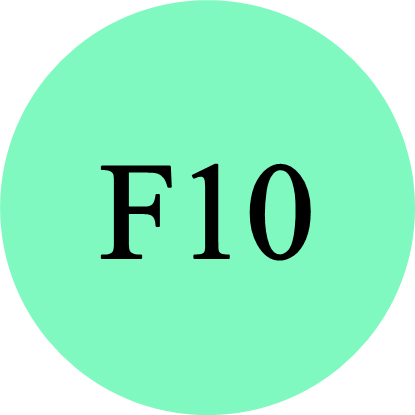

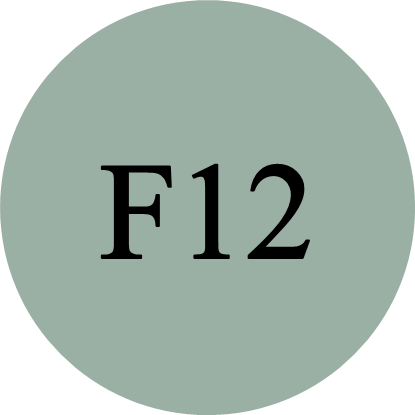

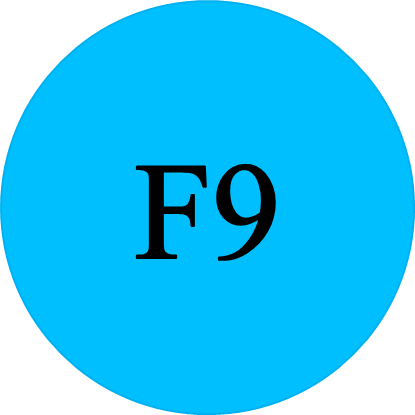

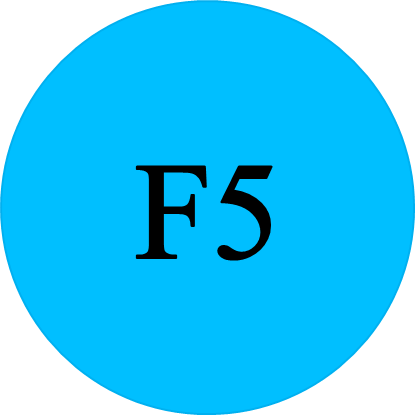

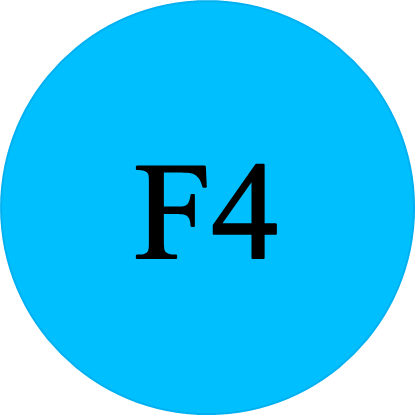

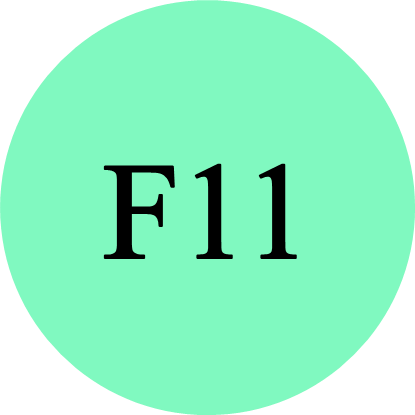

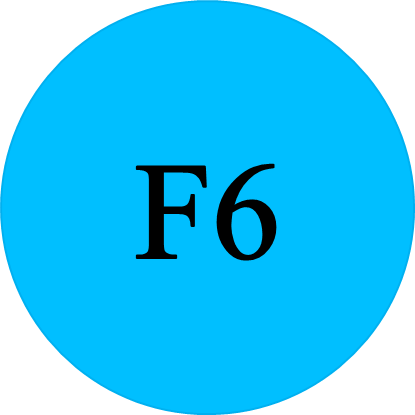

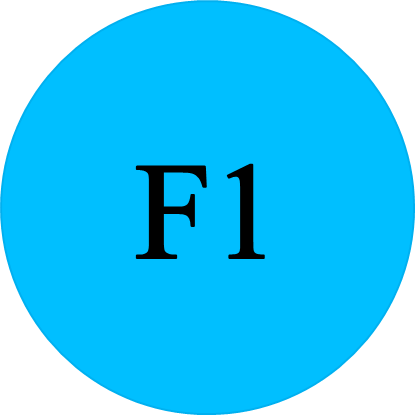

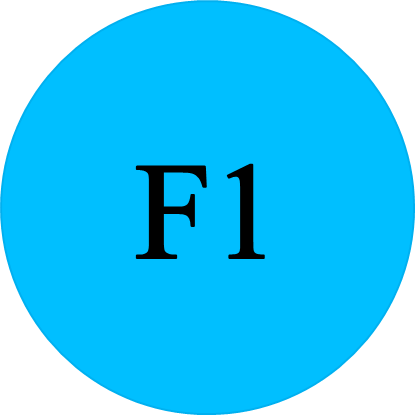


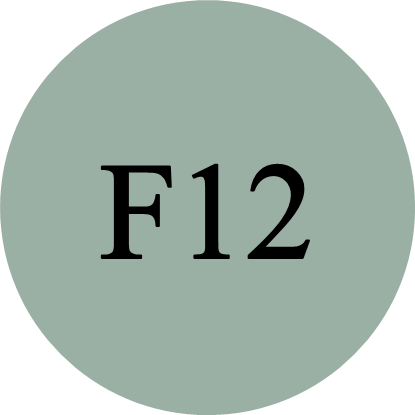

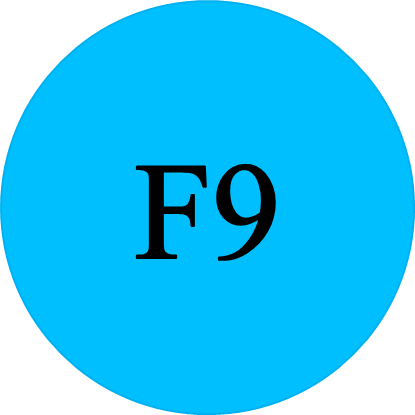

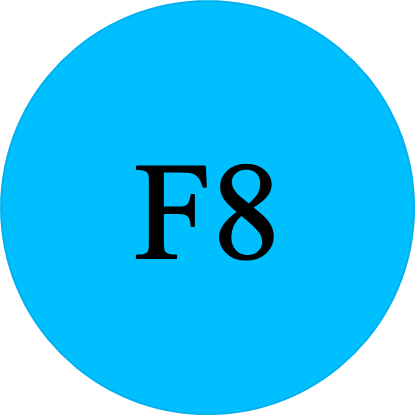

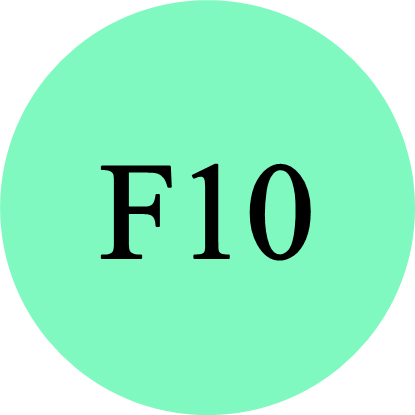

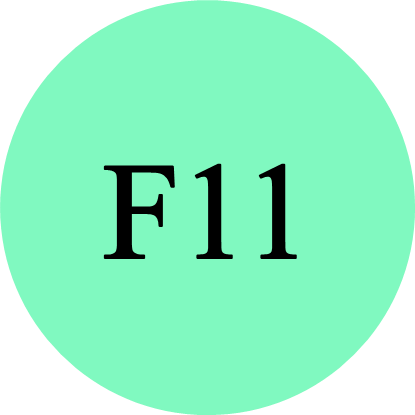

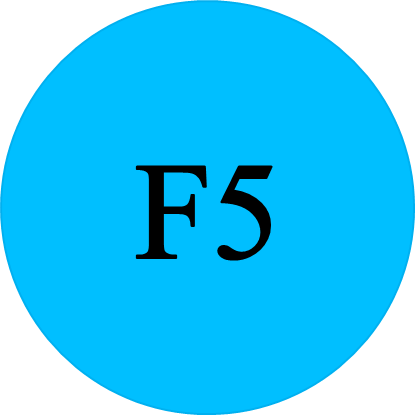

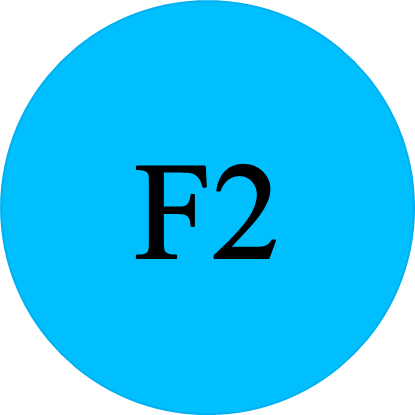

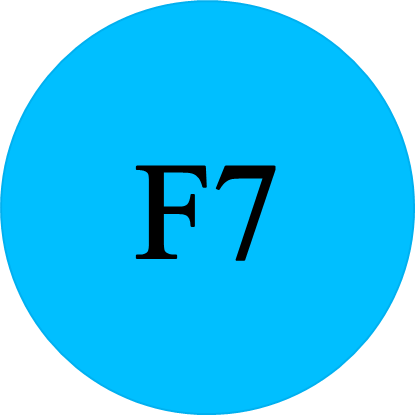

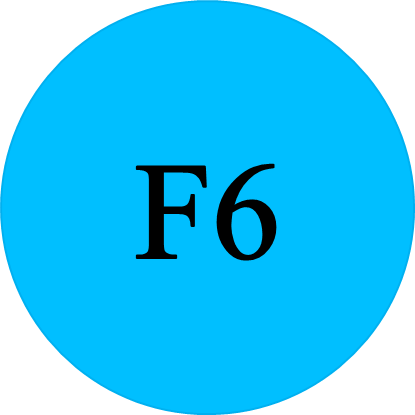

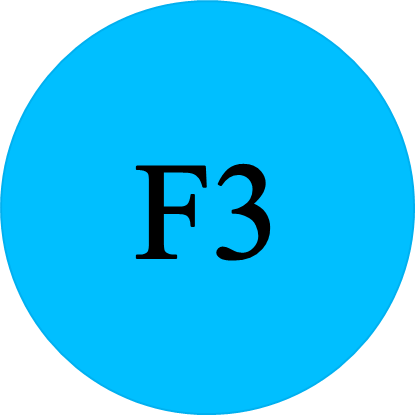

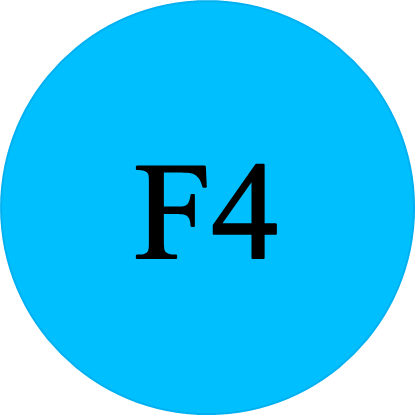

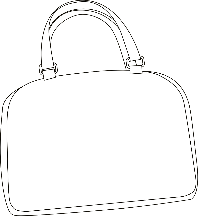

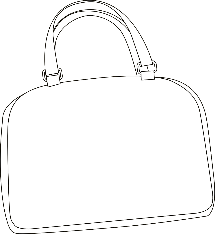

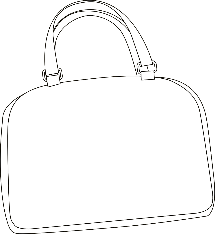
**Fig. S1** The schematic diagram of structure with random forest

……

……


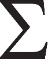


Output

Bagging

Decision trees

Dataset


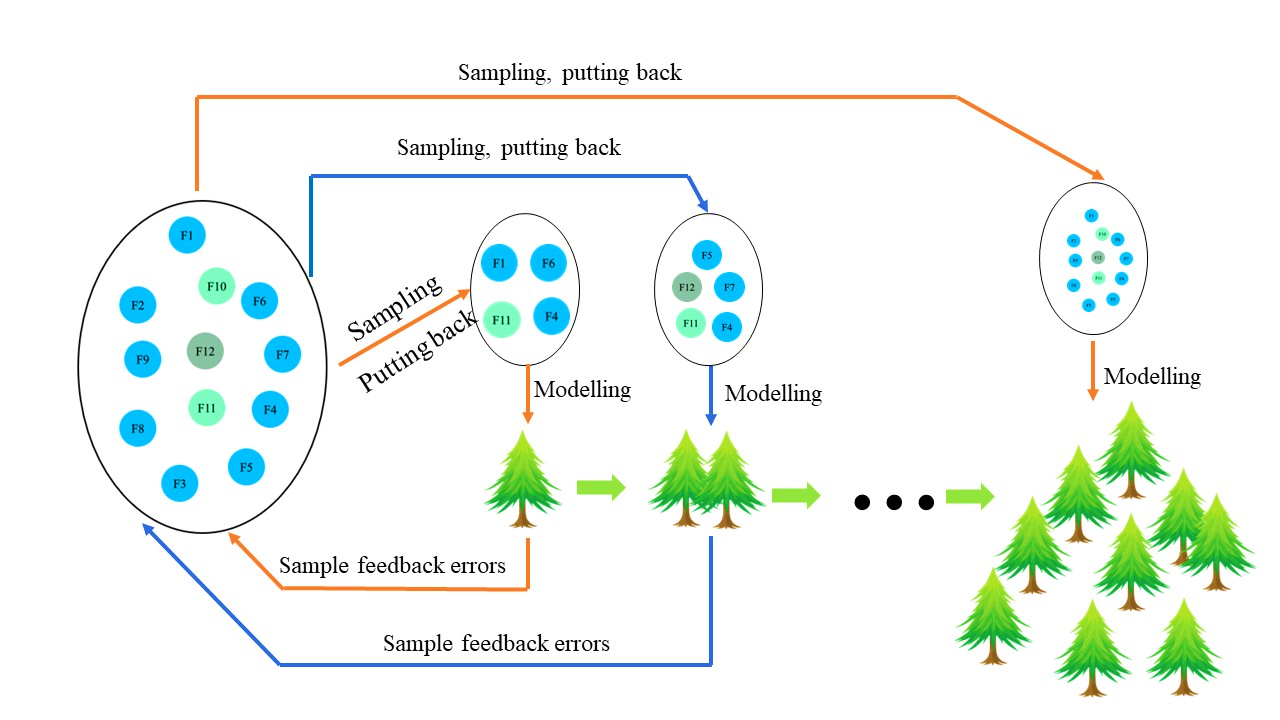


**Fig. S2.** Schematic diagram of structure with gradient boosting tree

**Table S1.** Details of biochars characteristics utilized in the study

|  | Biomass  feedstock | Pyrolysis temperature  ( ^o^C ) | Biochar characteristics | | | | | | | | | Reference |
| --- | --- | --- | --- | --- | --- | --- | --- | --- | --- | --- | --- | --- |
|  |  |  | pH_H2O_ | Total carbon  (%) | (O+N)/C | O/C | H/C | Ash  (%) | D (nm) | S(BET)(m^2^/g) | V (cm^3^/g) |  |
| 1 | Debarked loblolly pine chips(BC) | 300 | 5.08 | 56.4 | 0.668 | 0.67 | 0.1 | 1.5 | 24.4 | 1.4 | 0.008 | (Jang et al. 2018) |
| 2 | Activated debarked loblolly pine chips (ABC) | 300 | 6.83 | 86.8 | 0.16 | 0.16 | 0.0058 | 3.1 | 4.4 | 959.9 | 0.400 | (Jang et al., 2018) |
| 3 | Cow manure  (CMBC300) | 300 | 8.62 | 47.25 | 0.24 | 0.18 | 1.07 | 33.08 | 7.34 | 1.55 | 0.003 | (Zhang et al. 2019) |
| 4 | Cow manure  (CMBC500) | 500 | 10.75 | 43.08 | 0.17 | 0.13 | 0.45 | 45.55 | 5.89 | 1.77 | 0.003 | (Zhang et al., 2019) |
| 5 | Cow manure  (CMBC700) | 700 | 10.83 | 42.56 | 0.08 | 0.05 | 0.2 | 51.63 | 3 | 31.23 | 0.023 | (Zhang et al., 2019) |
| 6 | Rice straw  （R400） | 400 |  | 50.02 | 0.4 | 0.35 | 0.07 |  | 20.63 | 6.74 | 0.019 | (Wang et al. 2018) |
| 7 | Rice straw  （R600） | 600 |  | 55.33 | 0.22 | 0.2 | 0.03 |  | 17.04 | 21.69 | 0.05 | (Wang et al., 2018) |
| 8 | Swine manure  (M400) | 400 |  | 35.68 | 0.37 | 0.31 | 0.31 |  | 16.38 | 5.17 | 0.01 | (Wang et al., 2018) |
| 9 | Swine manure  (M600) | 600 |  | 31.27 | 0.31 | 0.25 | 0.25 |  | 12.36 | 10.56 | 0.04 | (Wang et al., 2018) |
| 10 | Maple leaves  （M-BC750） | 750 | 9.3 | 42.15 | 0.311 | 0.296 | 0.228 | 39.68 |  | 191.1 |  | (Kim et al. 2020) |
| 11 | Mesoporous batatas  （HMC-800） | 800 |  | 91.5 | 0.093 | 0.071 |  |  | 4.3 | 286.3 | 0.25 | (Zheng et al. 2021) |
| 12 | Wood-Based Panels（BC800） | 800 |  | 89.2 | 0.121 | 0.1 |  |  | 3.3 | 135.1 | 0.11 | (Xu et al. 2020) |
| 13 | Microalgae  （SPAL-BC350） | 350 | 8.3 | 66.6 | 0.259 | 0.138 | 0.138 | 10.43 |  | 2.63 |  | (Choi et al. 2020) |
| 14 | Microalgae  （SPAL-BC550） | 550 | 8.3 | 66.6 | 0.259 | 0.138 | 0.138 | 10.43 |  | 2.63 |  | (Choi et al., 2020) |
| 15 | Microalgae（SPAL-BC750） | 750 | 8.3 | 66.6 | 0.259 | 0.138 | 0.138 | 10.43 |  | 2.63 |  | (Choi et al., 2020) |
| 16 | Tea residue  （TWBC300） | 300 | 8.08 | 67.41 | 0.3229 | 0.2335 | 0.0774 | 5.6 |  | 1.991 |  | (Shisuo et al. 2020) |
| 17 | Tea residue  （TWBC500） | 500 | 8.08 | 67.41 | 0.3229 | 0.2335 | 0.0774 | 5.6 |  | 1.991 |  | (Shisuo et al., 2020) |
| 18 | Tea residue  （TWBC700） | 700 | 10.09 | 73.83 | 0.1967 | 0.12 | 0.0386 | 8.8 |  | 4.135 |  | (Shisuo et al., 2020) |
| 19 | Rice straw  （RCA） | 700 |  | 37.77 | 0.14 | 0.14 | 0.01 | 55.27 |  | 372.21 | 0.230 | (Chen et al. 2018) |
| 20 | Swine manure  （SCA） | 700 |  | 48.35 | 0.09 | 0.09 | 0.01 | 43.98 |  | 319.04 | 0.250 | (Chen et al., 2018) |
| 21 | Manganese dioxide modified herbal medicine residues（Mn-BC(1:10)） | 500 |  | 36.42 | 1.174 | 1.133 | 0.771 |  | 7.972 | 31.74 | 0.063 | (Shen et al. 2020) |
| 22 | Herbal medicine residues（H-BC） | 500 |  | 37.8 | 1.198 | 1.156 | 0.652 |  | 4.943 | 12.79 | 0.016 | (Shen et al., 2020) |

**Table S2.** The adsorption data of biochar for TC under different environmental conditions

|  | Biomass  feedstock | environmental conditions | | C_0_  (mmol/g) | Qe(mg/g) | Reference |
| --- | --- | --- | --- | --- | --- | --- |
|  |  | T (^o^C) | pH_sol_ |  |  |  |
| 1 | BC | 20 | 3.00 | 2.25 | 19.43 | (Jang et al., 2018) |
| 2 | BC | 20 | 5.00 | 2.25 | 25.15 |  |
| 3 | BC | 20 | 6.00 | 2.25 | 29.32 |  |
| 4 | BC | 20 | 7.00 | 2.25 | 18.17 |  |
| 5 | BC | 20 | 8.00 | 2.25 | 14.90 |  |
| 6 | BC | 20 | 9.00 | 2.25 | 9.55 |  |
| 7 | ABC | 20 | 3.00 | 2.25 | 243.98 |  |
| 8 | ABC | 20 | 5.00 | 2.25 | 247.25 |  |
| 9 | ABC | 20 | 6.00 | 2.25 | 277.27 |  |
| 10 | ABC | 20 | 7.00 | 2.25 | 261.22 |  |
| 11 | ABC | 20 | 8.00 | 2.25 | 189.81 |  |
| 12 | ABC | 20 | 9.00 | 2.25 | 179.11 |  |
| 13 | ABC | 20 | 6.00 | 0.22 | 99.16 |  |
| 14 | ABC | 20 | 6.00 | 0.45 | 171.31 |  |
| 15 | ABC | 20 | 6.00 | 0.67 | 218.00 |  |
| 16 | ABC | 20 | 6.00 | 0.90 | 238.87 |  |
| 17 | ABC | 20 | 6.00 | 1.12 | 247.97 |  |
| 18 | ABC | 20 | 6.00 | 1.35 | 257.07 |  |
| 19 | ABC | 20 | 6.00 | 1.57 | 266.72 |  |
| 20 | ABC | 20 | 6.00 | 1.80 | 274.35 |  |
| 21 | ABC | 20 | 6.00 | 2.02 | 279.49 |  |
| 22 | ABC | 20 | 6.00 | 2.25 | 275.91 |  |
| 23 | CMBC300 | 25 | 6.00 | 0.45 | 2.16 | (Zhang et al., 2019) |
| 24 | CMBC300 | 25 | 6.00 | 0.22 | 4.06 |  |
| 25 | CMBC300 | 25 | 6.00 | 0.15 | 4.31 |  |
| 26 | CMBC300 | 25 | 6.00 | 0.11 | 4.87 |  |
| 27 | CMBC300 | 25 | 6.00 | 0.09 | 5.18 |  |
| 28 | CMBC300 | 25 | 6.00 | 0.08 | 4.43 |  |
| 29 | CMBC300 | 25 | 6.00 | 0.06 | 4.90 |  |
| 30 | CMBC300 | 25 | 6.00 | 0.06 | 5.04 |  |
| 31 | CMBC500 | 25 | 6.00 | 0.45 | 16.67 |  |
| 32 | CMBC500 | 25 | 6.00 | 0.23 | 13.42 |  |
| 33 | CMBC500 | 25 | 6.00 | 0.15 | 11.06 |  |
| 34 | CMBC500 | 25 | 6.00 | 0.11 | 12.43 |  |
| 35 | CMBC500 | 25 | 6.00 | 0.09 | 9.80 |  |
| 36 | CMBC500 | 25 | 6.00 | 0.08 | 8.65 |  |
| 37 | CMBC500 | 25 | 6.00 | 0.06 | 9.22 |  |
| 38 | CMBC500 | 25 | 6.00 | 0.06 | 7.92 |  |
| 39 | CMBC700 | 25 | 6.00 | 0.45 | 26.00 |  |
| 40 | CMBC700 | 25 | 6.00 | 0.23 | 21.93 |  |
| 41 | CMBC700 | 25 | 6.00 | 0.15 | 15.19 |  |
| 42 | CMBC700 | 25 | 6.00 | 0.11 | 15.72 |  |
| 43 | CMBC700 | 25 | 6.00 | 0.09 | 13.63 |  |
| 44 | CMBC700 | 25 | 6.00 | 0.07 | 12.58 |  |
| 45 | CMBC700 | 25 | 6.00 | 0.06 | 11.21 |  |
| 46 | CMBC700 | 25 | 6.00 | 0.06 | 10.60 |  |
| 47 | CMBC300 | 25 | 3.00 | 0.09 | 3.48 |  |
| 48 | CMBC300 | 25 | 4.00 | 0.09 | 4.12 |  |
| 49 | CMBC300 | 25 | 5.00 | 0.09 | 4.89 |  |
| 50 | CMBC300 | 25 | 6.00 | 0.09 | 5.23 |  |
| 51 | CMBC300 | 25 | 7.00 | 0.09 | 6.20 |  |
| 52 | CMBC300 | 25 | 8.00 | 0.09 | 12.52 |  |
| 53 | CMBC300 | 25 | 9.00 | 0.09 | 14.34 |  |
| 54 | CMBC300 | 25 | 10.00 | 0.09 | 11.62 |  |
| 55 | CMBC500 | 25 | 3.00 | 0.09 | 12.35 |  |
| 56 | CMBC500 | 25 | 4.00 | 0.09 | 12.87 |  |
| 57 | CMBC500 | 25 | 5.00 | 0.09 | 10.76 |  |
| 58 | CMBC500 | 25 | 6.00 | 0.09 | 9.83 |  |
| 59 | CMBC500 | 25 | 7.00 | 0.09 | 5.23 |  |
| 60 | CMBC500 | 25 | 8.00 | 0.09 | 6.73 |  |
| 61 | CMBC500 | 25 | 9.00 | 0.09 | 3.98 |  |
| 62 | CMBC500 | 25 | 10.00 | 0.09 | 2.43 |  |
| 63 | CMBC700 | 25 | 3.00 | 0.09 | 18.90 |  |
| 64 | CMBC700 | 25 | 4.00 | 0.09 | 16.88 |  |
| 65 | CMBC700 | 25 | 5.00 | 0.09 | 14.77 |  |
| 66 | CMBC700 | 25 | 6.00 | 0.09 | 13.60 |  |
| 67 | CMBC700 | 25 | 7.00 | 0.09 | 7.75 |  |
| 68 | CMBC700 | 25 | 8.00 | 0.09 | 7.90 |  |
| 69 | CMBC700 | 25 | 9.00 | 0.09 | 8.07 |  |
| 70 | CMBC700 | 25 | 10.00 | 0.09 | 7.55 |  |
| 71 | CMBC300 | 25 | 6.00 | 0.02 | 1.92 |  |
| 72 | CMBC300 | 25 | 6.00 | 0.04 | 3.60 |  |
| 73 | CMBC300 | 25 | 6.00 | 0.05 | 4.04 |  |
| 74 | CMBC300 | 25 | 6.00 | 0.07 | 5.22 |  |
| 75 | CMBC300 | 25 | 6.00 | 0.09 | 5.97 |  |
| 76 | CMBC300 | 25 | 6.00 | 0.11 | 7.44 |  |
| 77 | CMBC300 | 25 | 6.00 | 0.13 | 7.92 |  |
| 78 | CMBC300 | 25 | 6.00 | 0.14 | 10.02 |  |
| 79 | CMBC500 | 25 | 6.00 | 0.02 | 4.86 |  |
| 80 | CMBC500 | 25 | 6.00 | 0.04 | 5.58 |  |
| 81 | CMBC500 | 25 | 6.00 | 0.05 | 6.60 |  |
| 82 | CMBC500 | 25 | 6.00 | 0.07 | 7.26 |  |
| 83 | CMBC500 | 25 | 6.00 | 0.09 | 8.40 |  |
| 84 | CMBC500 | 25 | 6.00 | 0.11 | 10.11 |  |
| 85 | CMBC500 | 25 | 6.00 | 0.13 | 11.01 |  |
| 86 | CMBC500 | 25 | 6.00 | 0.14 | 12.99 |  |
| 87 | CMBC700 | 25 | 6.00 | 0.02 | 5.28 |  |
| 88 | CMBC700 | 25 | 6.00 | 0.04 | 6.57 |  |
| 89 | CMBC700 | 25 | 6.00 | 0.05 | 8.16 |  |
| 90 | CMBC700 | 25 | 6.00 | 0.07 | 9.27 |  |
| 91 | CMBC700 | 25 | 6.00 | 0.09 | 9.93 |  |
| 92 | CMBC700 | 25 | 6.00 | 0.11 | 13.23 |  |
| 93 | CMBC700 | 25 | 6.00 | 0.13 | 14.49 |  |
| 94 | CMBC700 | 25 | 6.00 | 0.14 | 16.89 |  |
| 95 | R400 | 25 | 2.01 | 0.01 | 2.41 | (Wang et al., 2018) |
| 96 | R400 | 25 | 3.50 | 0.01 | 3.10 |  |
| 97 | R400 | 25 | 5.00 | 0.01 | 3.47 |  |
| 98 | R400 | 25 | 6.51 | 0.01 | 3.08 |  |
| 99 | R400 | 25 | 8.02 | 0.01 | 2.85 |  |
| 100 | R400 | 25 | 9.51 | 0.01 | 1.93 |  |
| 101 | R400 | 25 | 11.03 | 0.01 | 1.38 |  |
| 102 | R600 | 25 | 1.99 | 0.01 | 3.37 |  |
| 103 | R600 | 25 | 3.49 | 0.01 | 3.69 |  |
| 104 | R600 | 25 | 4.99 | 0.01 | 4.57 |  |
| 105 | R600 | 25 | 6.51 | 0.01 | 4.30 |  |
| 106 | R600 | 25 | 8.01 | 0.01 | 3.76 |  |
| 107 | R600 | 25 | 9.52 | 0.01 | 2.49 |  |
| 108 | R600 | 25 | 11.01 | 0.01 | 2.05 |  |
| 109 | M400 | 25 | 2.02 | 0.01 | 1.80 |  |
| 110 | M400 | 25 | 3.50 | 0.01 | 2.60 |  |
| 111 | M400 | 25 | 4.99 | 0.01 | 2.85 |  |
| 112 | M400 | 25 | 6.53 | 0.01 | 2.69 |  |
| 113 | M400 | 25 | 8.00 | 0.01 | 2.37 |  |
| 114 | M400 | 25 | 9.52 | 0.01 | 1.42 |  |
| 115 | M400 | 25 | 11.02 | 0.01 | 1.14 |  |
| 116 | M600 | 25 | 1.99 | 0.01 | 2.40 |  |
| 117 | M600 | 25 | 3.50 | 0.01 | 3.05 |  |
| 118 | M600 | 25 | 5.02 | 0.01 | 3.30 |  |
| 119 | M600 | 25 | 6.49 | 0.01 | 3.20 |  |
| 120 | M600 | 25 | 8.01 | 0.01 | 3.08 |  |
| 121 | M600 | 25 | 9.53 | 0.01 | 2.19 |  |
| 122 | M600 | 25 | 11.02 | 0.01 | 1.74 |  |
| 123 | M-BC750 | 20 | 3.00 | 2.25 | 115.49 | (Kim et al., 2020) |
| 124 | M-BC750 | 20 | 4.01 | 2.25 | 282.45 |  |
| 125 | M-BC750 | 20 | 5.01 | 2.25 | 330.37 |  |
| 126 | M-BC750 | 20 | 5.99 | 2.25 | 334.46 |  |
| 127 | M-BC750 | 20 | 7.02 | 2.25 | 299.98 |  |
| 128 | M-BC750 | 20 | 8.02 | 2.25 | 173.86 |  |
| 129 | M-BC750 | 20 | 9.03 | 2.25 | 76.33 |  |
| 130 | M-BC750 | 20 | 6.00 | 0.22 | 60.03 |  |
| 131 | M-BC750 | 20 | 6.00 | 0.45 | 79.41 |  |
| 132 | M-BC750 | 20 | 6.00 | 0.67 | 112.22 |  |
| 133 | M-BC750 | 20 | 6.00 | 1.12 | 198.51 |  |
| 134 | M-BC750 | 20 | 6.00 | 1.35 | 229.26 |  |
| 135 | M-BC750 | 20 | 6.00 | 1.57 | 304.96 |  |
| 136 | M-BC750 | 20 | 6.00 | 1.80 | 304.19 |  |
| 137 | M-BC750 | 20 | 6.00 | 2.02 | 344.88 |  |
| 138 | M-BC750 | 20 | 6.00 | 2.25 | 366.33 |  |
| 139 | HMC-800 | 25 | 7.00 | 0.25 | 73.04 | (Zheng et al., 2021) |
| 140 | HMC-800 | 25 | 7.00 | 0.08 | 56.67 |  |
| 141 | HMC-800 | 25 | 7.00 | 0.05 | 43.55 |  |
| 142 | HMC-800 | 25 | 7.00 | 0.04 | 24.09 |  |
| 143 | HMC-800 | 25 | 7.00 | 0.03 | 12.05 |  |
| 144 | HMC-800 | 25 | 7.00 | 0.10 | 37.88 |  |
| 145 | HMC-800 | 25 | 7.00 | 0.22 | 56.59 |  |
| 146 | HMC-800 | 25 | 7.00 | 0.44 | 70.55 |  |
| 147 | HMC-800 | 25 | 7.00 | 0.67 | 86.56 |  |
| 148 | HMC-800 | 25 | 7.00 | 1.13 | 118.90 |  |
| 149 | HMC-800 | 25 | 7.00 | 2.25 | 145.99 |  |
| 150 | HMC-800 | 25 | 7.00 | 3.38 | 183.50 |  |
| 151 | HMC-800 | 25 | 7.00 | 4.50 | 193.10 |  |
| 152 | HMC-800 | 25 | 3.02 | 0.22 | 40.46 |  |
| 153 | HMC-800 | 25 | 5.03 | 0.22 | 49.08 |  |
| 154 | HMC-800 | 25 | 7.06 | 0.22 | 56.12 |  |
| 155 | HMC-800 | 25 | 9.03 | 0.22 | 55.61 |  |
| 156 | HMC-800 | 25 | 11.05 | 0.22 | 49.71 |  |
| 157 | HMC-800 | 15 | 7.00 | 0.11 | 31.29 |  |
| 158 | HMC-800 | 15 | 7.00 | 0.22 | 45.21 |  |
| 159 | HMC-800 | 15 | 7.00 | 0.45 | 55.80 |  |
| 160 | HMC-800 | 15 | 7.00 | 0.67 | 66.10 |  |
| 161 | HMC-800 | 15 | 7.00 | 1.12 | 84.09 |  |
| 162 | HMC-800 | 15 | 7.00 | 2.25 | 111.04 |  |
| 163 | HMC-800 | 15 | 7.00 | 3.37 | 126.22 |  |
| 164 | HMC-800 | 15 | 7.00 | 4.50 | 130.30 |  |
| 165 | HMC-800 | 25 | 7.00 | 0.11 | 39.76 |  |
| 166 | HMC-800 | 25 | 7.00 | 0.22 | 58.06 |  |
| 167 | HMC-800 | 25 | 7.00 | 0.45 | 70.31 |  |
| 168 | HMC-800 | 25 | 7.00 | 0.67 | 87.87 |  |
| 169 | HMC-800 | 25 | 7.00 | 1.12 | 119.23 |  |
| 170 | HMC-800 | 25 | 7.00 | 2.25 | 146.54 |  |
| 171 | HMC-800 | 25 | 7.00 | 3.37 | 184.47 |  |
| 172 | HMC-800 | 25 | 7.00 | 4.50 | 193.03 |  |
| 173 | HMC-800 | 35 | 7.00 | 0.11 | 44.26 |  |
| 174 | HMC-800 | 35 | 7.00 | 0.22 | 67.50 |  |
| 175 | HMC-800 | 35 | 7.00 | 0.45 | 90.31 |  |
| 176 | HMC-800 | 35 | 7.00 | 0.67 | 122.48 |  |
| 177 | HMC-800 | 35 | 7.00 | 1.12 | 144.54 |  |
| 178 | HMC-800 | 35 | 7.00 | 2.25 | 173.91 |  |
| 179 | HMC-800 | 35 | 7.00 | 3.37 | 221.58 |  |
| 180 | HMC-800 | 35 | 7.00 | 4.50 | 237.52 |  |
| 181 | BC800 | 25 | 7.00 | 0.03 | 5.84 | (Xu et al., 2020) |
| 182 | BC800 | 25 | 7.00 | 0.02 | 5.11 |  |
| 183 | BC800 | 25 | 7.00 | 0.02 | 4.91 |  |
| 184 | BC800 | 25 | 7.00 | 0.01 | 4.24 |  |
| 185 | BC800 | 25 | 7.00 | 0.01 | 3.86 |  |
| 186 | BC800 | 25 | 7.00 | 0.00 | 0.99 |  |
| 187 | BC800 | 25 | 7.00 | 0.00 | 1.98 |  |
| 188 | BC800 | 25 | 7.00 | 0.01 | 3.81 |  |
| 189 | BC800 | 25 | 7.00 | 0.02 | 4.83 |  |
| 190 | BC800 | 25 | 7.00 | 0.03 | 5.23 |  |
| 191 | BC800 | 25 | 7.00 | 0.04 | 5.68 |  |
| 192 | BC800 | 25 | 7.00 | 0.04 | 6.09 |  |
| 193 | BC800 | 25 | 7.00 | 0.05 | 6.60 |  |
| 194 | BC800 | 25 | 2.00 | 0.02 | 7.24 |  |
| 195 | BC800 | 25 | 3.00 | 0.02 | 6.62 |  |
| 196 | BC800 | 25 | 4.00 | 0.02 | 5.32 |  |
| 197 | BC800 | 25 | 5.00 | 0.02 | 5.13 |  |
| 198 | BC800 | 25 | 6.00 | 0.02 | 5.10 |  |
| 199 | BC800 | 25 | 7.00 | 0.02 | 4.96 |  |
| 200 | BC800 | 25 | 8.00 | 0.02 | 5.13 |  |
| 201 | BC800 | 25 | 9.00 | 0.02 | 5.26 |  |
| 202 | BC800 | 25 | 10.00 | 0.02 | 5.35 |  |
| 203 | BC800 | 15 | 7.00 | 0.02 | 4.95 |  |
| 204 | BC800 | 25 | 7.00 | 0.02 | 5.75 |  |
| 205 | BC800 | 35 | 7.00 | 0.02 | 6.78 |  |
| 206 | SPAL-BC350 | 20 | 6.00 | 2.25 | 14.92 | (Choi et al., 2020) |
| 207 | SPAL-BC550 | 20 | 6.00 | 2.25 | 80.38 |  |
| 208 | SPAL-BC750 | 20 | 6.00 | 2.25 | 132.04 |  |
| 209 | SPAL-BC750 | 20 | 6.00 | 2.25 | 131.91 |  |
| 210 | SPAL-BC750 | 30 | 6.00 | 2.25 | 231.43 |  |
| 211 | SPAL-BC750 | 40 | 6.00 | 2.25 | 520.37 |  |
| 212 | SPAL-BC750 | 20 | 3.00 | 2.25 | 103.98 |  |
| 213 | SPAL-BC750 | 20 | 4.00 | 2.25 | 97.62 |  |
| 214 | SPAL-BC750 | 20 | 5.00 | 2.25 | 132.30 |  |
| 215 | SPAL-BC750 | 20 | 6.00 | 2.25 | 131.06 |  |
| 216 | SPAL-BC750 | 20 | 7.00 | 2.25 | 167.90 |  |
| 217 | SPAL-BC750 | 20 | 8.00 | 2.25 | 275.50 |  |
| 218 | SPAL-BC750 | 20 | 9.00 | 2.25 | 278.45 |  |
| 219 | SPAL-BC750 | 20 | 6.00 | 0.22 | 62.84 |  |
| 220 | SPAL-BC750 | 20 | 6.00 | 0.45 | 95.82 |  |
| 221 | SPAL-BC750 | 20 | 6.00 | 0.67 | 104.71 |  |
| 222 | SPAL-BC750 | 20 | 6.00 | 0.90 | 123.50 |  |
| 223 | SPAL-BC750 | 20 | 6.00 | 1.12 | 136.96 |  |
| 224 | SPAL-BC750 | 20 | 6.00 | 1.35 | 133.61 |  |
| 225 | SPAL-BC750 | 20 | 6.00 | 1.57 | 134.09 |  |
| 226 | SPAL-BC750 | 20 | 6.00 | 2.02 | 140.10 |  |
| 227 | SPAL-BC750 | 20 | 6.00 | 2.25 | 136.74 |  |
| 228 | TWBC300 | 25 | 8.00 | 0.04 | 1.31 | (Shisuo et al., 2020) |
| 229 | TWBC300 | 25 | 8.00 | 0.02 | 1.09 |  |
| 230 | TWBC300 | 25 | 8.00 | 0.01 | 0.48 |  |
| 231 | TWBC300 | 25 | 8.00 | 0.01 | 0.39 |  |
| 232 | TWBC300 | 25 | 8.00 | 0.01 | 0.20 |  |
| 233 | TWBC500 | 25 | 10.00 | 0.05 | 4.50 |  |
| 234 | TWBC500 | 25 | 10.00 | 0.02 | 5.06 |  |
| 235 | TWBC500 | 25 | 10.00 | 0.01 | 2.95 |  |
| 236 | TWBC500 | 25 | 10.00 | 0.01 | 2.28 |  |
| 237 | TWBC500 | 25 | 10.00 | 0.01 | 1.91 |  |
| 238 | TWBC700 | 25 | 10.00 | 0.05 | 7.95 |  |
| 239 | TWBC700 | 25 | 10.00 | 0.02 | 5.79 |  |
| 240 | TWBC700 | 25 | 10.00 | 0.01 | 3.93 |  |
| 241 | TWBC700 | 25 | 10.00 | 0.01 | 2.89 |  |
| 242 | TWBC700 | 25 | 10.00 | 0.01 | 2.32 |  |
| 243 | TWBC700 | 25 | 3.01 | 0.01 | 2.27 |  |
| 244 | TWBC700 | 25 | 5.01 | 0.01 | 2.28 |  |
| 245 | TWBC700 | 25 | 7.00 | 0.01 | 2.27 |  |
| 246 | TWBC700 | 25 | 9.01 | 0.01 | 2.29 |  |
| 247 | TWBC700 | 25 | 11.02 | 0.01 | 2.28 |  |
| 248 | RCA | 25 | 7.00 | 0.27 | 95.87 | (Chen et al., 2018) |
| 249 | RCA | 25 | 7.00 | 0.36 | 104.44 |  |
| 250 | RCA | 25 | 7.00 | 0.45 | 105.50 |  |
| 251 | RCA | 25 | 7.00 | 0.54 | 116.85 |  |
| 252 | RCA | 25 | 7.00 | 0.72 | 130.32 |  |
| 253 | RCA | 25 | 7.00 | 0.90 | 141.17 |  |
| 254 | RCA | 25 | 7.00 | 1.35 | 148.88 |  |
| 255 | RCA | 25 | 7.00 | 1.80 | 152.35 |  |
| 256 | SCA | 25 | 7.00 | 0.27 | 104.44 |  |
| 257 | SCA | 25 | 7.00 | 0.36 | 116.68 |  |
| 258 | SCA | 25 | 7.00 | 0.45 | 120.88 |  |
| 259 | SCA | 25 | 7.00 | 0.54 | 136.80 |  |
| 260 | SCA | 25 | 7.00 | 0.72 | 145.21 |  |
| 261 | SCA | 25 | 7.00 | 0.90 | 156.92 |  |
| 262 | SCA | 25 | 7.00 | 1.35 | 162.68 |  |
| 263 | SCA | 25 | 7.00 | 1.80 | 164.07 |  |
| 264 | H-BC | 25 | 7.00 | 0.53 | 4.45 | (Shen et al., 2020) |
| 265 | H-BC | 25 | 7.00 | 0.27 | 7.00 |  |
| 266 | H-BC | 25 | 7.00 | 0.11 | 5.57 |  |
| 267 | H-BC | 25 | 7.00 | 0.07 | 6.13 |  |
| 268 | H-BC | 25 | 7.00 | 0.06 | 6.72 |  |
| 269 | H-BC | 25 | 7.00 | 0.05 | 5.57 |  |
| 270 | Mn-BC(1:10) | 25 | 6.00 | 0.04 | 5.29 |  |
| 271 | Mn-BC(1:10) | 25 | 6.00 | 0.54 | 85.59 |  |
| 272 | Mn-BC(1:10) | 25 | 6.00 | 0.28 | 67.41 |  |
| 273 | Mn-BC(1:10) | 25 | 6.00 | 0.11 | 34.02 |  |
| 274 | Mn-BC(1:10) | 25 | 6.00 | 0.07 | 22.35 |  |
| 275 | Mn-BC(1:10) | 25 | 6.00 | 0.06 | 19.11 |  |
| 276 | Mn-BC(1:10) | 25 | 6.00 | 0.05 | 16.28 |  |
| 277 | Mn-BC(1:10) | 25 | 6.00 | 0.04 | 13.48 |  |
| 278 | H-BC | 25 | 2.00 | 0.28 | 12.95 |  |
| 279 | H-BC | 25 | 3.00 | 0.28 | 3.71 |  |
| 280 | H-BC | 25 | 4.00 | 0.28 | 2.91 |  |
| 281 | H-BC | 25 | 5.00 | 0.28 | 9.22 |  |
| 282 | H-BC | 25 | 6.00 | 0.28 | 8.66 |  |
| 283 | H-BC | 25 | 7.00 | 0.28 | 7.21 |  |
| 284 | H-BC | 25 | 8.00 | 0.28 | 11.74 |  |
| 285 | H-BC | 25 | 9.00 | 0.28 | 19.52 |  |
| 286 | H-BC | 25 | 10.00 | 0.28 | 21.13 |  |
| 287 | Mn-BC(1:10) | 25 | 2.00 | 0.28 | 76.61 |  |
| 288 | Mn-BC(1:10) | 25 | 3.00 | 0.28 | 80.49 |  |
| 289 | Mn-BC(1:10) | 25 | 4.00 | 0.28 | 64.94 |  |
| 290 | Mn-BC(1:10) | 25 | 5.00 | 0.28 | 66.69 |  |
| 291 | Mn-BC(1:10) | 25 | 6.00 | 0.28 | 68.29 |  |
| 292 | Mn-BC(1:10) | 25 | 7.00 | 0.28 | 71.25 |  |
| 293 | Mn-BC(1:10) | 25 | 8.00 | 0.28 | 73.26 |  |
| 294 | Mn-BC(1:10) | 25 | 9.00 | 0.28 | 67.63 |  |
| 295 | Mn-BC(1:11) | 25 | 10.00 | 0.28 | 61.06 |  |

**Table S3.** Molecular structures and physiochemical properties of TC

|  | Tetracycline (TC) |
| --- | --- |
| Molecular structure | 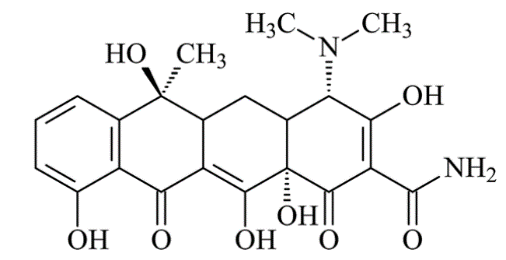 |
| Molecular weight (g/mol) | 444.4 |
| Molecular volume (cm^3^/mol) | 266.3±7.0 |
| pK_a_ | pK_1_ = 3.3, pK_2_ = 7.7, pK_3_ = 9.7 |
| Antibiotics species at different pH | 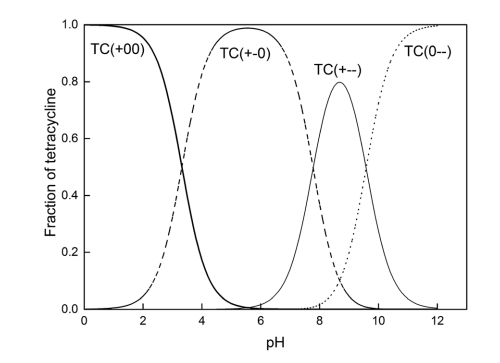(Wang et al., 2018) |

**Table S4.** Machine Learning Model Parameters and Results

|  | Random forest (RF) | Gradient Boosting Decision Tree (GBDT) | XGBoost | Artificial Neural Network (ANN) |
| --- | --- | --- | --- | --- |
| Theoretical basis | Ensemble machine learning method by averaging the performance of decision trees | Ensemble ML method by constantly reducing the prediction errors of decision trees (boosting theory) | Let the gradient tree increase to the limit of its own computing power. | ANNs simulate brain neurons' connections and signal transmission, using nodes and weights to achieve complex pattern recognition and nonlinear data transformation. |
| Input parameters  {range of values，interval} | n_estimators = {0-400,25}  max_depth = {5-60,5}  max_leaf_nodes = {25-50,5}  min_samples_split = {2-6,1}  min_samples_leaf = {1-5,1} | n_estimators = {0-400, 25}  max_depth = {5-60, 5}  max_leaf_nodes = {25-50,5}  min_samples_split = {2-5,1}  min_samples_leaf ={1-5,1} | n_estimators = {0-400, 25}  max_depth = {5-60, 5}  learning_rate = [0.001,0.01,0.1,0.2,0.3]  subsample = [0.1, 0.5, 0.8, 1]  gamma =[0, 0.1, 0.5,1] | hidden layer = {5-30, 5}  activation function for hidden layer = ‘sigmoid’  learning rate = [0.001,0.01,0.1,0.2,0.3] |
| Output parameters | (i) The value of predicted adsorption efficiency in test group  (ii) R^2^ between predicted and actual values  (iii) Root mean squared error (RMSE) between predicted and actual values | (i) The value of predicted adsorption efficiency in test group  (ii) R^2^ between predicted and actual values  (iii) Root mean squared error (RMSE) between predicted and actual values | (i) The value of predicted adsorption efficiency in test group  (ii) R^2^ between predicted and actual values  (iii) Root mean squared error (RMSE) between predicted and actual values | (i) The value of predicted adsorption efficiency in test group  (ii) R^2^ between predicted and actual values  (iii) Root mean squared error (RMSE) between predicted and actual values |
| Best parameters | n_estimators=300  max_depth=45  max_leaf_nodes=45  min_samples_split=2  min_samples_leaf=1 | n_estimators = 50  max_depth = 10  max_leaf_nodes = 35  min_samples_split = 3  min_samples_leaf = 2 | max_depth = 10  n_estimators = 100  learning_rate = 0.2  subsample = 1  gamma = 0 | hidden layer = 30  learning rate = 0.01 |
| Results | R^2^ = 0.9625  RMSE = 18.02 | R^2^ = 0.9152  RMSE = 25.97 | R^2^ = 0.9592  RMSE = 20.99 | R^2^ = 0.9410  RMSE = 22.42 |

**Table S5.** Statistical values of biochar characteristic parameters

|  | pH_H2O | C | (O+N)/C | O/C | H/C | Ash | D | S(BET) | V |
| --- | --- | --- | --- | --- | --- | --- | --- | --- | --- |
| count | 152 | 295 | 295 | 295 | 228 | 168 | 221 | 295 | 237 |
| mean | 9.142829 | 59.98461 | 0.300858 | 0.261992 | 0.338289 | 29.84036 | 6.98624 | 140.9825 | 0.111225 |
| std | 1.545797 | 21.48974 | 0.327511 | 0.325406 | 0.339692 | 18.89268 | 5.175129 | 228.4262 | 0.122989 |
| min | 5.08 | 31.27 | 0.08 | 0.05 | 0.0058 | 1.5 | 3 | 1.4 | 0.002612 |
| 25% | 8.3 | 42.56 | 0.107 | 0.0835 | 0.07 | 10.2725 | 4.3 | 2.63 | 0.013 |
| 50% | 9.3 | 48.35 | 0.17 | 0.138 | 0.2 | 33.08 | 4.943 | 31.23 | 0.054 |
| 75% | 10.75 | 86.8 | 0.31 | 0.25 | 0.45 | 45.55 | 7.34 | 238.7 | 0.249 |
| max | 10.83 | 91.5 | 1.198 | 1.156 | 1.07 | 55.27 | 24.4 | 959.9 | 0.4 |

**Reference**

Chen T, Luo L, Deng S, Shi G, Zhang S, Zhang Yet alWei L (2018) Sorption of tetracycline on H3PO4 modified biochar derived from rice straw and swine manure. Bioresour Technol. 267: 431-437. <https://doi.org/10.1016/j.biortech.2018.07.074>

Choi Y-K, Choi T-R, Gurav R, Bhatia S K, Park Y-L, Kim H Jet alYang Y-H (2020) Adsorption behavior of tetracycline onto Spirulina sp.(microalgae)-derived biochars produced at different temperatures. Sci Total Environ. 710: 136282. <https://doi.org/10.1016/j.scitotenv.2019.136282>

Jang H M, Yoo S, Choi Y-K, Park S and Kan E (2018) Adsorption isotherm, kinetic modeling and mechanism of tetracycline on Pinus taeda-derived activated biochar. Bioresour Technol. 259: 24-31. <https://doi.org/10.1016/j.biortech.2018.03.013>

Kim J E, Bhatia S K, Song H J, Yoo E, Jeon H J, Yoon J-Yet alKim H J (2020) Adsorptive removal of tetracycline from aqueous solution by maple leaf-derived biochar. Bioresour Technol. 306: 123092. <https://doi.org/10.1016/j.biortech.2020.123092>

Shen Q, Wang Z, Yu Q, Cheng Y, Liu Z, Zhang T and Zhou S (2020) Removal of tetracycline from an aqueous solution using manganese dioxide modified biochar derived from Chinese herbal medicine residues. Environ Res. 183: 109195. <https://doi.org/10.1016/j.envres.2020.109195>

Shisuo F, Wenpu L, Jintao W, Hongmei H, Yining Y and Na Z (2020) Preparation of tea residue biochar and its removal characteristics of tetracycline in solution. Environmental Science. 41: 1308-1318. 10.13227/j.hjkx.201908179

Wang H, Fang C, Wang Q, Chu Y, Song Y, Chen Y and Xue X (2018) Sorption of tetracycline on biochar derived from rice straw and swine manure. RSC advances. 8: 16260-16268. 10.1039/C8RA01454J

Xu D, Gao Y, Lin Z, Gao W, Zhang H, Karnowo Ket alZhang S (2020) Application of biochar derived from pyrolysis of waste fiberboard on tetracycline adsorption in aqueous solution. Frontiers in chemistry. 7: 943. <https://doi.org/10.3389/fchem.2019.0094>

Zhang P, Li Y, Cao Y and Han L (2019) Characteristics of tetracycline adsorption by cow manure biochar prepared at different pyrolysis temperatures. Bioresour Technol. 285: 121348. <https://doi.org/10.1016/j.biortech.2019.121348>

Zheng Z, Zhao B, Guo Y, Guo Y, Pak T and Li G (2021) Preparation of mesoporous batatas biochar via soft-template method for high efficiency removal of tetracycline. Sci Total Environ. 787: 147397. <https://doi.org/10.1016/j.scitotenv.2021.147397>
